# Multi‐omics analysis identifies PUS7 as an immune modulator driving NETs‐mediated macrophage polarization in pancreatic cancer

**DOI:** 10.1002/ctm2.70581

**Published:** 2026-01-06

**Authors:** Jike Fang, Shiye Ruan, Yajie Wang, Yue Chen, Fuxin Huang, Zhongyan Zhang, Chuanzhao Zhang, Baohua Hou, Shanzhou Huang

**Affiliations:** ^1^ Department of General Surgery Guangdong Provincial People's Hospital, Guangdong Academy of Medical Sciences, Southern Medical University Guangzhou China; ^2^ The Second School of Clinical Medicine Southern Medical University Guangzhou China; ^3^ South China University of Technology School of Medicine Guangzhou China; ^4^ Division of Hepatobiliary and Pancreas Surgery Department of General Surgery Shenzhen People's Hospital (The Second Clinical Medical College, Jinan University; The First Affiliated Hospital, Southern University of Science and Technology) Shenzhen China; ^5^ Maoming People's Hospital Maoming China

**Keywords:** macrophage polarization, neutrophil extracellular traps, pancreatic ductal adenocarcinoma, PUS family, PUS7, tumour microenvironment

## Abstract

Pseudouridine synthases (PUS) have been implicated in various cancers, yet their roles in pancreatic cancer immunity remain unclear. Through integrative multi‐omics analyses combining genomics, transcriptomics, and clinical datasets, we evaluated associations between PUS family genes and oncogenic features, including tumour microenvironment scores, immune infiltration, cancer stemness, and prognosis. Among them, PUS7 and PUS3 showed the strongest correlations with tumour‐promoting phenotypes, with high PUS7 expression in PDAC predicting poor overall survival. Functional assays revealed that PUS7 overexpression markedly enhanced PDAC cell proliferation, migration, and invasion. Transcriptomic profiling demonstrated that PUS7 promotes neutrophil extracellular traps formation, identifying it as a key regulator of NET‐mediated immune modulation. Single‐cell RNA sequencing of orthotopic mouse models showed PUS7 overexpression reduced macrophage infiltration and skewed macrophage polarization towards the M2 phenotype while suppressing M1 polarization. We found that PUS7 reshapes the PDAC immune landscape primarily by inducing NETs, which drive macrophage polarization from M1 to M2, fostering immune suppression and tumour progression. The PUS7–NET–M2/M1 axis thus represents a novel mechanism of PDAC pathogenesis and a potential therapeutic target in this lethal malignancy.

**Highlights:**

- PUS7 enhances pancreatic ductal adenocarcinoma progression by modulating the tumour immune microenvironment.
- PUS7 induces neutrophil extracellular trap formation within pancreatic tumours.
- NETs reprogram tumour‐associated macrophages from an M1 to an M2 phenotype, promoting immune suppression.

## Graphical abstract

PUS7 reshapes the PDAC immune landscape primarily by inducing NETs, which drive macrophage polarization from M1 to M2, fostering immune suppression and tumour progression. The inhibitory effects of S428‐1145 and DNase I on pancreatic cancer are mediated through blocking the PUS7/NETs/M2 macrophage pathway.

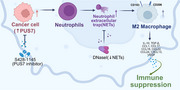

## 1 INTRODUCTION

Pancreatic ductal adenocarcinoma (PDAC) is one of the most lethal malignancies worldwide, ranking 12th in incidence but as high as 6th in cancer‐related mortality.^1^ Currently, surgical resection remains the only potentially curative treatment; however, only approximately 20% of patients are eligible for surgery at the time of diagnosis. Although immunotherapies such as PD‐1 immune checkpoint inhibitors have achieved breakthrough success in some malignancies,^2^ their efficacy in PDAC has been significantly limited. This is primarily attributed to the highly immunosuppressive tumour microenvironment (TME) of PDAC, characterized by extensive stromal fibrosis and infiltration of immunosuppressive cells, including cancer‐associated fibroblasts (CAFs), tumour‐associated macrophages (TAMs), and exhausted cytotoxic T lymphocytes.^3^ This immunological barrier not only promotes tumour growth and metastasis but also severely impedes the clinical success of immunotherapy.^4^

RNA modifications have emerged as critical regulators of gene expression and tumour immunity. Among them, pseudouridine (Ψ) is one of the most abundant modifications in RNA and is catalyzed by pseudouridine synthases, which isomerize uridine to pseudouridine.^5^, ^6^ The human genome encodes 13 pseudouridine synthases (PUS), classified into five evolutionarily conserved families: TruA, TruB, TruD, RluA, and Pus10p.^7^ These enzymes are broadly involved in RNA splicing, stability, and translational regulation, and their dysregulation has been implicated in various human diseases. The regulation of RNA splicing, stability, and translation by PUS enzymes is primarily achieved through their catalysis of pseudouridine (Ψ) formation.^8^ The PUS family has been increasingly recognized for its involvement in cancer and other human diseases. Among them, PUS7—the sole member of the TruD family has been linked to tumour progression in glioblastoma, colorectal cancer, and gastric cancer, with both oncogenic and tumour‐suppressive roles reported depending on the cancer type.^9^, ^10^, ^11^, ^12^ In addition, other PUS family members, such as PUS1, PUS3, RPUSD3, RPUSD4, and PUS10, have been associated with mitochondrial dysfunction, neurodevelopmental disorders, and autoimmune diseases.^13^, ^14^, ^15^, ^16^ The five families of PUS enzymes are functionally distinguished based on their conserved catalytic domains and substrate specificities. PUS7, the focus of this study, may differ in substrate selection from members of other families. These findings suggest that PUS enzymes may participate in diverse biological processes and contribute to disease development in a context‐dependent manner.

Neutrophil extracellular traps (NETs) are web‐like structures composed of DNA, histones, proteases, and antimicrobial proteins, released by neutrophils through a series of processes involving reactive oxygen species, neutrophil elastase (NE), myeloperoxidase (MPO) activity, and citrullinated histone H3 (CitH3).^17^ Initially described as antimicrobial defence structures, NETs have since emerged as important contributors to tumour progression. Studies have demonstrated that NETs can promote epithelial–mesenchymal transition,^18^ enhance tumour growth in ovarian cancer,^19^ and accelerate PDAC progression in murine models.^20^ Beyond their direct effects on tumour cells, NETs also shape the tumour immune microenvironment. For example, they can activate pancreatic stellate cells to support tumour proliferation,^21^ and have been shown to induce M2 polarization of TAMs in melanoma^22^ and gastric cancer,^23^ thereby facilitating immune evasion and metastasis.

In this study, we performed a comprehensive pan‐cancer analysis of the PUS family, integrating transcriptomic, genomic, epigenetic, and clinical datasets to systematically evaluate their expression patterns, prognostic relevance, pathway associations, and relationships with the TME across diverse cancer types. Through this integrative framework, we sought to identify PUS family members with potential context‐dependent roles in tumour progression and immune regulation.

Notably, although PUS family genes exhibited heterogeneous expression and clinical associations across multiple malignancies, PDAC emerged as a tumour type in which PUS‐related signatures showed particularly strong links to immune suppression–related features of the TME. Given the defining characteristics of PDAC—including its dense stroma, profound immune exclusion, and resistance to immune checkpoint blockade—this cancer type represents a biologically relevant context in which dysregulated RNA modification may exert pronounced immunomodulatory effects.

In parallel, accumulating evidence has established NETs as critical mediators of immune evasion and disease progression in PDAC. These observations led us to hypothesize that aberrant activity of specific PUS enzymes may contribute to PDAC pathogenesis by modulating NET formation and downstream immune cell polarization. Therefore, PDAC was selected as a focused model to investigate the potential role of PUS7 in coordinating RNA modification–dependent immune remodelling within the tumour microenvironment.

## 2 MATERIALS AND METHODS

### 2.1 Data acquisition and preprocessing

TCGA data for pan cancer analysis, including gene expression profiles, clinicopathological features, molecular subtypes, survival outcomes, immune infiltration subtypes, and tumour stemness indices, were obtained and analyzed using the UCSC Xena platform (https://xenabrowser.net/).^24^ For the TCGA‐PAAD cohort, after excluding (pseudo) normal tissues and non‐PDAC tumour samples, a total of 150 primary PDAC tumour samples were included for further analysis.^25^ Data on tumour‐infiltrating immune cells were retrieved from the TIMER2 database (http://timer.cistrome.org/). All datasets underwent strict preprocessing, including removal of low‐quality samples, missing values, Z‐score normalization, and batch effect correction to ensure data robustness and consistency. In addition, external validation of PUS7 expression was performed using the GEO datasets GSE15471 and GSE62165 (https://www.ncbi.nlm.nih.gov/geo/).^26^, ^27^

### 2.2 Expression landscape and co‐expression analysis of PUS family genes across cancers

First, transcriptomic data from 33 cancer types in the TCGA database were filtered to exclude normal tissue samples, retaining only tumour tissue expression profiles. A boxplot was generated to visualize the overall expression patterns of the 11 PUS family members across cancers. Subsequently, for 18 TCGA cancer types with more than five adjacent normal tissues (NT) samples, differential expression analyses were performed to compare PUS gene expression between tumour tissues (CT) and adjacent normal tissues. The Wilcoxon rank‐sum test was applied to assess expression differences and paired trends. In addition, co‐expression patterns among PUS family members were systematically evaluated using the ‘corrplot’ package in R, revealing potential coordinated expression relationships within the gene family.

### 2.3 Somatic copy number alteration and mutation analysis

To investigate the relationship between genomic alterations and mRNA expression levels of PUS family genes, Spearman correlation analysis was performed based on the GSCA platform (Genome Sequence Cancer Analysis, http://bioinfo.life.hust.edu.cn/GSCA/#/).^28^ GSCA is an integrated and interactive resource that enables comprehensive visualization and analysis of multi‐omics data, particularly focusing on the association between genomic alterations and immune‐related features in cancer. Using GSCA, we systematically analyzed various genomic alterations in PUS family genes, including single‐nucleotide variations (SNVs), copy number variations (CNVs), and DNA methylation patterns. For DNA methylation analysis, only cancer types with more than 10 paired tumour and adjacent normal tissue samples were included to ensure reliability.

In addition, the cBioPortal database (https://www.cbioportal.org/)^29^ was utilized to further evaluate the mutation frequency and spectrum of PUS family genes across large‐scale cancer cohorts, including TCGA and ICGC datasets. cBioPortal integrates multi‐omics and clinical data from over 28 000 tumour samples, providing a widely used resource for comprehensive cancer genomics studies.

### 2.4 Cox regression analysis of PUS family gene expression and prognostic significance in pan‐cancer

Univariate Cox proportional hazards regression models were applied to systematically evaluate the association between the expression levels of individual PUS family members and patient prognosis across multiple cancer types. The survival endpoints included overall survival (OS), disease‐specific survival (DSS), and progression‐free survival (PFS). All analyses were performed using the ‘survival’ package in R. Forest plots were generated with the ‘forestplot’ package to visually present the hazard ratios (HRs) and corresponding 95% confidence intervals (CIs), providing an intuitive assessment of the potential value of PUS family genes as prognostic biomarkers in pan‐cancer.

### 2.5 Immune infiltration subtypes analysis

To evaluate immune infiltration patterns within the TME, the association between PUS family gene expression and six immune subtypes (C1–C6) was analyzed using an ANOVA model.^30^ Statistical significance was assessed using the Wilcoxon rank‐sum test, and the results were visualized with the ‘ggplot2’ package in R.

### 2.6 Construction of the PUS scoring system

A PUS scoring system was established using the single‐sample Gene Set Enrichment Analysis (ssGSEA) algorithm implemented in the ‘GSVA’ package in R. For each sample across 33 TCGA cancer types, ssGSEA ranked all genes based on their expression levels and calculated an enrichment score based on the relative expression of the PUS gene set present in the dataset. Specifically, the ssGSEA algorithm first ranks genes for each sample, then calculates an enrichment score by summing the expression ranks of the PUS family genes. The final PUS score (PUSs) for each sample was derived from the empirical cumulative distribution function of the permuted scores.

### 2.7 Survival analysis based on the PUS score

To assess the prognostic significance of the PUSs, univariate Cox proportional hazards regression analysis was performed across 33 cancer types using the ‘limma,’ ‘survival,’ and ‘survminer’ packages in R. The association between the PUSs and patient survival outcomes was systematically evaluated to explore its potential as a prognostic value across pan‐cancer cohorts.

### 2.8 TME, stemness indices, and immune checkpoints in pan‐cancer

To comprehensively evaluate the association between the PUSs, PUS family gene expression, and TME characteristics, we calculated the stromal score, immune score, ESTIMATE score, and tumour purity for each patient across 33 cancer types using the “ESTIMATE” package in R. These metrics reflect the levels of immune cell infiltration and stromal content within tumour tissues. Subsequently, the correlations between the PUSs and TME‐related indices were assessed using Spearman correlation analysis implemented via the ‘cor.test,’ ‘ggpubr,’ and ‘limma’ packages in R. The results were visualized as heatmaps using the ‘ggplot2’ package. Additionally, Spearman correlation tests were used to explore the relationship between PUSs and tumour mutational burden (TMB) as well as microsatellite instability (MSI) across 33 cancer types. Correlation results between PUSs and DNAss, RNAss, TMB, and MSI were visualized using radar plots generated by the ‘fmsb’ package. A total of 47 immune checkpoint‐related genes were obtained from previously published studies.^31^ The correlations between these immune checkpoint genes, the PUSs, and the expression of individual PUS family members were systematically analyzed.

### 2.9 GSVA‐based pathway enrichment analysis of PUSs

To explore the potential biological functions and pan‐cancer regulatory mechanisms of PUSs, Gene Set Variation Analysis (GSVA) was performed using the ‘GSVA’ package in R^32^ across 33 cancer types. The analysis assessed the correlation between the PUSs and 50 classical HALLMARK signalling pathways. The HALLMARK gene sets were obtained from the MSigDB database (http://software.broadinstitute.org/gsea/msigdb/index.jsp),^33^ covering key biological processes such as tumourigenesis, cell proliferation, immune response, and metabolic reprogramming.

### 2.10 Correlation analysis between PUS7 expression and drug sensitivity based on the CellMiner database

Drug sensitivity and gene expression data were obtained from the CellMiner database (https://discover.nci.nih.gov/cellminer/home.do). A systematic analysis was conducted to evaluate the correlation between PUS7 expression levels and the sensitivity to various anticancer drugs. During data processing, missing values were imputed and preprocessed using the ‘impute’ and ‘limma’ packages in R. Statistical analysis and visualization were performed with the ‘ggplot2’ and ‘ggpubr’ packages, aiming to explore the potential impact of PUS7 expression on tumour cell drug responsiveness.

### 2.11 Screening by AutoDock‐GPU

AutoDock Vina and DoGSiteScorer were used to preprocess the protein to generate the docking grid file and construct the pharmacophore model of the small molecule compound database. The 300 000 compounds library was obtained from ChemDiv. AutoDock‐GPU were used to calculate the docking score. The top six small molecules with the highest scores were applied for the subsequent MD simulation.

### 2.12 Molecular dynamics simulations

The docking results were selected as the initial structure. The Gromacs 2025 version was used as the dynamics simulation software. Small molecule preprocessing was performed using AmberTools22 to apply the GAFF force field. Gaussian 16 W was used for hydrogenation and calculation of RESP potentials. The Amber99sb‐ildn force field was employed, with water molecules as the solvent TIP3P model, and the total charge was neutralized sodium ion equilibrium system. The canonical system (NVT) and isothermal isobaric system (NPT) equilibrium systems are used. Finally, a free molecular dynamics simulation was run for a total of 100 ns after the completion of the simulation. The root mean square deviation (RMSD), root mean square rise and fall (RMSF), and protein radius of gyration (Rg) for each amino acid trajectory, combined with the free energy (MMGBSA), Hbond, solvent‐accessible surface area (SASA) and the free energy landscape, were analyzed using the software's built‐in tools.

### 2.13 Cellular thermal shift assay

Drugs were obtained from Shanghai Topscience Limited Corporation (Shanghai, China). The powder was stored at −20°C and dissolved with DMSO for cellular thermal shift assay (CETSA). Cell lysates were incubated with the indicated molecule for 2 h at 37°C. 30 µg total protein were heated at different temperatures (37, 40, 43, 46, 49, 52, 55, and 58°C) for 3 min and centrifuged at 12 000 rpm, 4°C for 15 min. The protein level of PUS7 was tested by Westernblot.

### 2.14 Clinical specimens

Between January 2019 and December 2022, paired tumour and adjacent non‐tumour tissue samples (*n* = 56) were obtained from PDAC patients undergoing surgical resection at Guangdong Provincial People's Hospital. None of the patients had received any antitumour therapy before surgery. Written informed consent was obtained from all participants.

### 2.15 Cell culture

The HPDE, AsPC‐1, BxPC‐3, PANC‐1, MIA PaCa‐2, CAPAN‐2, SW1990, and THP‐1 cell lines used in this study were obtained from Procell and confirmed to be free of mycoplasma contamination. Cell cultures were maintained following standard procedures. Briefly, cells were cultured in DMEM or RPMI 1640 medium (Gibco), supplemented with 10% fetal bovine serum (FBS, Gibco), and incubated at 37°C in a humidified atmosphere containing 5% CO_2_. Stably transfected cell lines were maintained under selection using medium containing 2.5–5 µg/mL puromycin. All experiments were performed with cells that had undergone fewer than 20 passages.

### 2.16 Plasmids and lentiviral packaging

Plasmids for target gene overexpression were constructed by MiaoLing Bio (Wuhan, China), and short hairpin RNAs (shRNAs) for gene knockdown were designed and synthesized by RiboBio (Guangzhou, China). The expression plasmid for PUS7 overexpression was cloned into the psin‐EF1α vector. shRNAs targeting human or murine PUS7 were subcloned into the PLKO.1 vector. The PUS7 knockdown shRNA sequences are shown in Table S1. Lentiviruses were produced by co‐transfecting HEK293T cells with the expression or shRNA plasmids along with the packaging plasmids pMD2.G and psPAX2. The supernatants were collected and used to infect target cells. Stable PUS7‐overexpressing or knockdown cell lines were established via puromycin selection.

### 2.17 Quantitative real‐time PCR

Total RNA was extracted using the FastPure cell/tissue total RNA isolation kit (RC112‐01, Vazyme). Quantitative real‐time PCR (qRT‐PCR) was performed using the 5× Evo M‐MLV RT Master Mix (AG1706, Accurate Biology) for reverse transcription and the 2× SYBR Green Pro Taq HS Premix (AG11735, Accurate Biology) for amplification. Primer sequences are listed in Table S2. Relative gene expression levels were calculated using the 2^−ΔΔCt^ method.

### 2.18 Western blot

Total protein was extracted using RIPA lysis buffer (Beyotime, China), and protein concentrations were determined using the BCA protein assay kit (Beyotime). 20 µg total protein were loaded onto SDS‐PAGE gels and transferred onto polyvinylidene difluoride (PVDF) membranes (Bio‐Rad). Membranes were blocked with 5% non‐fat milk for 1 h at room temperature, followed by incubation with primary antibodies. After washing, membranes were incubated with HRP‐conjugated secondary antibodies. Protein bands were visualized using an enhanced chemiluminescence (ECL) detection system (Thermo Fisher Scientific). Details of the primary antibodies used are provided in Tables S3 and S4.

### 2.19 Construction of animal models

To establish a PDAC xenograft model, PANC‐1 cells were subcutaneously injected into the flanks of nude mice (1 × 10^7^ cells in 100 µL PBS per mouse). Tumour growth was monitored weekly, and tumour volume was calculated using the formula: Volume (cm^3^) = (length × width^2^)/2. After 4 weeks, the mice were euthanized, and tumours were harvested and weighed.

An orthotopic pancreatic tumour model was established in C57BL/6 mice using Panc02 cells expressing empty vector or PUS7 cDNA. Briefly, a small incision was made in the left abdominal flank of the mice near the spleen, and 20 µL cell suspension was injected into the pancreas using a sterile syringe. Tumour formation and body weight changes were monitored every 7 days. All animal procedures were approved by the Animal Ethics Committee of Guangdong Provincial People's Hospital and conducted under specific pathogen‐free (SPF) conditions with controlled temperature and humidity, a 12 h light/dark cycle, and access to standard chow. Mice were sacrificed at 4 weeks post‐injection, and tumour weights were measured.

### 2.20 Wound healing assay

PANC‐1 and MIA PaCa‐2 cells (5 × 10^6^) were seeded into six‐well plates. Upon reaching approximately 90% confluence, a uniform scratch (∼1 mm in width) was introduced using a sterile 200 µL pipette tip. The wounded area was imaged under a microscope every 24 h to monitor cell migration.

### 2.21 Colony formation assay

PANC‐1 and MIA PaCa‐2 cells were transduced with lentiviruses for PUS7 overexpression or knockdown, followed by selection with 5 µg/mL puromycin to establish stable cell lines. Approximately 1000 stably transduced PANC‐1 and MIA PaCa‐2 cells, along with their respective control cells, were seeded into six‐well plates. After two weeks of incubation, cell colonies were fixed with 4% paraformaldehyde for 30 min and stained with 0.5% crystal violet (Solarbio, Beijing, China).

### 2.22 5‐Ethynyl‐20‐deoxyuridine assay

PANC‐1 and MIA PaCa‐2 pancreatic cancer cells with stable PUS7 overexpression (OE), knockdown (KD), or empty vector controls (Vector) were seeded in 35‐mm glass‐bottom confocal dishes (5 × 10^3^ cells/dish). After 24 h culture, proliferation was assessed using the BeyoClick EdU Kit with DAB (Beyotime, C0085S). Cells were pulse‐labelled with 10 µM 5‐ethynyl‐20‐deoxyuridine (EdU) for 2 h at 37°C, fixed in 4% PFA (15 min, RT), and permeabilized with 0.3% Triton X‐100 (15 min, RT). Click reaction was performed using a freshly prepared mixture (Click Reaction Buffer, CuSO_4_, Biotin Azide, and Click Additive Solution; 500 µL/dish, 30 min, room temperature [RT], dark). After washing, samples were incubated with Streptavidin‐HRP (1:100, 30 min, RT) followed by DAB chromogenic development (1:1 mixture of Solutions A/B, 5–10 min) until brown nuclear staining appeared. EdU^+^ nuclei were imaged via brightfield mode on a Zeiss LSM 880 confocal microscope (20× objective).

### 2.23 Transwell assay

Cell migration and invasion abilities were assessed using 24‐well Transwell chambers with or without Matrigel coating. Briefly, PANC‐1 and MIA PaCa‐2 cells stably transduced with PUS7‐overexpressing or PUS7‐knockdown lentiviruses were selected using 5 µg/mL puromycin and seeded into the upper chambers in serum‐free medium. Complete medium containing serum was added to the lower chambers as a chemoattractant. After incubation at 37°C with 5% CO_2_ for 24 h, cells on the lower surface of the membrane were fixed with 4% paraformaldehyde for 20 min and stained with 0.1% crystal violet (Solarbio). Migrated or invaded cells were then visualized and quantified under a fluorescence microscope.

### 2.24 Cell viability and IC_50_ determination

MIA PaCa‐2 and PANC‐1 cells were seeded in 96‐well plates at 3 × 10^3^ cells/well and allowed to attach overnight. Cells were treated with S428‐1145 at increasing concentrations (0, 5, 10, 15, 20, 25, 30, 35, and 40 µM) for 72 h. Cell viability was measured using the CCK‐8 assay according to the manufacturer's instructions. IC_50_ values were calculated using GraphPad Prism 10 by fitting a nonlinear regression model.

### 2.25 RNA‐seq

Total RNA was assessed using the Agilent 2100 Bioanalyzer. mRNA was enriched via Oligo(dT) magnetic beads, fragmented, and reverse‐transcribed to synthesize double‐stranded cDNA. After purification, end‐repair, A‐tailing, and adapter ligation, libraries were size‐selected, PCR‐amplified, and quality‐checked. Raw reads were filtered to obtain clean reads, which were aligned to the reference genome for transcript assembly, novel transcript prediction, and splicing analysis. Protein‐coding transcripts were added to build a reference transcriptome. Gene expression was quantified by FPKM using StringTie, and differential expression was analyzed with Ballgown. FDR‐adjusted *p*‐values < .05 were considered significant.

### 2.26 Immunohistochemistry

The sections were first deparaffinized and rehydrated, followed by antigen retrieval using EDTA solution in a steamer. Endogenous peroxidase activity was blocked with 3% hydrogen peroxide. The sections were then incubated overnight at 4°C with a primary antibody. On the following day, the sections were washed with PBS and incubated with a secondary antibody for 1 h. DAB and hematoxylin were used for light counterstaining. Immunohistochemical (IHC) staining results were independently scored by two investigators blinded to group assignments, followed by semi‐quantitative analysis using predefined criteria detailed in Table S5.^34^ The inter‐observer agreement between the two pathologists for the semi‐quantitative H‐score was assessed using Cohen's kappa (κ) statistic. A κ value greater than .80 was considered to represent excellent agreement.

### 2.27 Immunofluorescence assay

Cells were fixed with 4% paraformaldehyde at RT for 10 min, followed by washing with PBS. Permeabilization and blocking were performed using 2% bovine serum albumin (BSA) and 0.5% Triton X‐100 at RT for 30 min. After PBS washing, cells were incubated overnight at 4°C with either anti‐MPO and anti‐Histone H3 antibodies (1:100 dilution). Subsequently, the cells were washed and incubated with 0.1 µg/mL DAPI (62248, Invitrogen) at RT for 1 h. Images were acquired using a confocal laser scanning microscope (Zeiss). Details of the primary antibodies used are provided in Table S3.

### 2.28 Macrophage polarization assay

Cells were cultured under identical conditions for 24 h, after which the conditioned media were collected. THP‐1 cells were treated with phorbol 12‐myristate 13‐acetate (PMA, 100 ng/mL) for 24 h to induce differentiation into M0 macrophages. Cells were then washed with PBS and cultured in the collected conditioned media for an additional 48 h under the same incubation conditions. Following treatment, cells were harvested and stained with the primary antibodies. The expression levels of CD86 and CD163 were subsequently analyzed by flow cytometry.

### 2.29 Flow cytometry

Tumours were excised from euthanized mice, mechanically dissociated, and digested with Collagenase IV (Sigma) at 37°C for 30 min. The resulting cell suspension was filtered through a 70 µm cell strainer, washed twice with PBS, and then subjected to flow cytometric staining using the primary antibodies. Stained cells were analyzed on a BD FACSymphony flow cytometer (BD Biosciences), and data were processed using FlowJo software.

### 2.30 DNase I treatment regimen in C57BL/6 mice

Recombinant DNase I (Sigma‐Aldrich) was dissolved in sterile PBS immediately before use. C57BL/6 mice received DNase I at a dose of 100 U per mouse, administered intraperitoneally (i.p.) once daily. Treatment was initiated when tumours became palpable and continued daily throughout the experimental period (typically 10–21 days). Control mice received an equal volume of sterile PBS following the same schedule.

### 2.31 Quantification of NETosis‐positive cells by fluorescence imaging

To quantify the percentage of neutrophils undergoing NETosis, live‐cell dual‐fluorescence imaging was performed. Isolated neutrophils were co‐stained with Hoechst 33342 (for total nuclei) and SYTOX Green (for extracellular DNA) and imaged under physiological conditions (37°C, 5% CO_2_) using a fluorescence microscope. Image analysis was conducted with ImageJ. The total cell number per field was counted based on the Hoechst signal. A cell was defined as NETosis‐positive if it concurrently exhibited (1) a decondensed, cloud‐like nuclear morphology and (2) a SYTOX Green signal intensity ≥threefold above background in that region. The percentage of NETosis‐positive cells was calculated as (positive cells/total cells) × 100% for each field and averaged across biological replicates.

### 2.32 Single‐cell RNA sequencing data processing and analysis

Raw data from the Illumina NovaSeq6000 platform were demultiplexed and converted to FASTQ format using bcl2fastq (v2.19.0.316). The cDNA reads were then aligned to the mouse reference genome (mm10). Using Cell Ranger (v6.1.2), we performed quality control to filter low‐quality cells and genes, counted barcodes and UMIs, and generated a filtered gene‐cell matrix. Subsequent analysis was performed with the Seurat R package (v3.2.3), involving further filtering, log‐normalization of gene expression, identification of highly variable genes, and principal component analysis (PCA) for dimensionality reduction. Data integration across samples was achieved using the Harmony R package. Cell clusters were annotated by matching cluster‐specific marker genes with known cell‐type signatures from the literature and the CellMarker database. To ensure neutrophils were not inadvertently excluded, we annotated cell clusters following dimensionality reduction and clustering using classical neutrophil markers (such as S100A8 and S100A9), and did not pre‐filter any cell populations.

### 2.33 Statistical analysis

In this study, statistical analyses were conducted using SPSS software version 26.0 (IBM, Armonk, NY), GraphPad Prism version 10.0 (GraphPad Software, La Jolla, CA), and R software version 4.3.6 (AT&T Bell Laboratories).

## 3 RESULTS

### 3.1 Characterization of PUS family in pan‐cancer analysis

**FIGURE 1:**
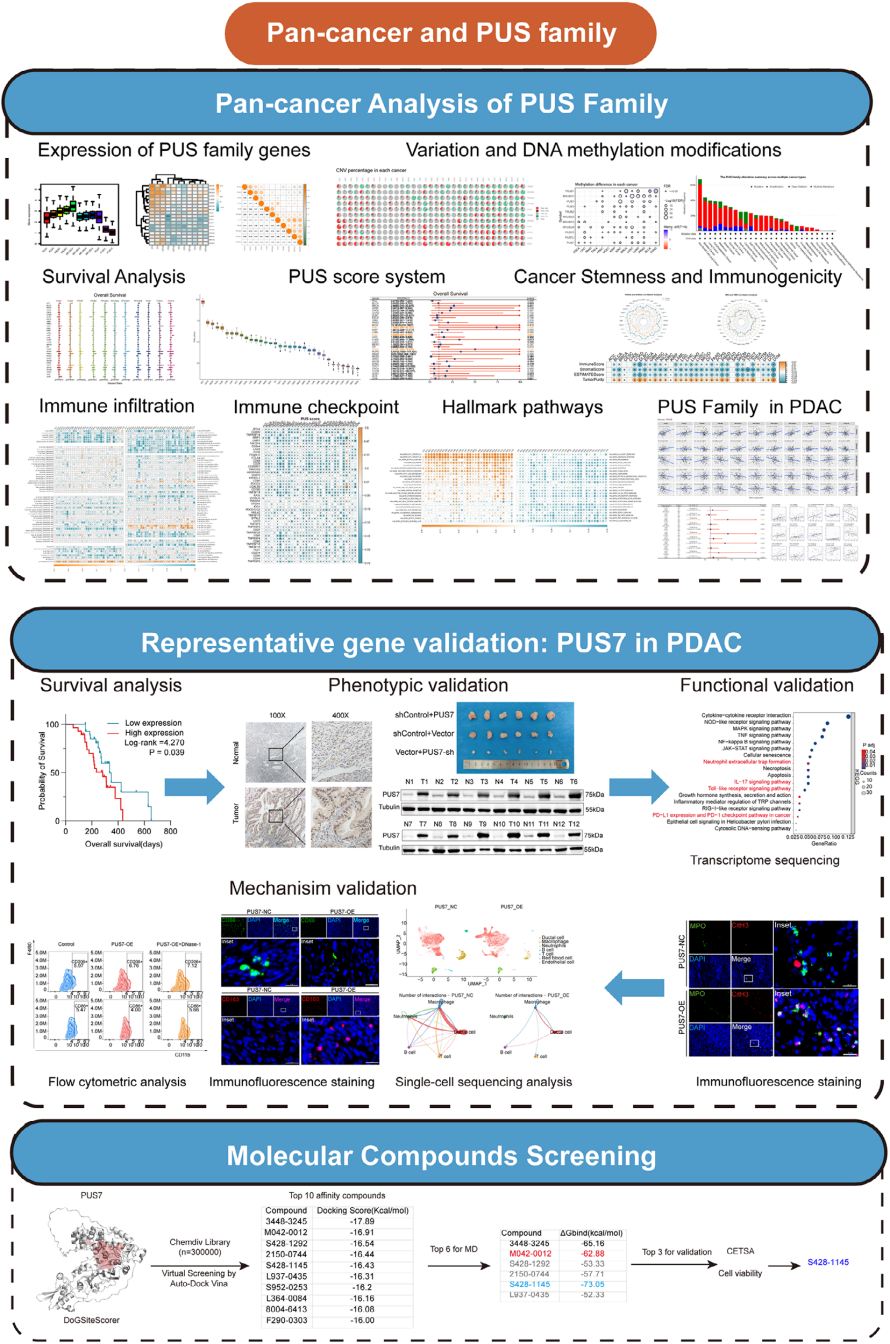
Flow diagram of the work.

**FIGURE 2:**
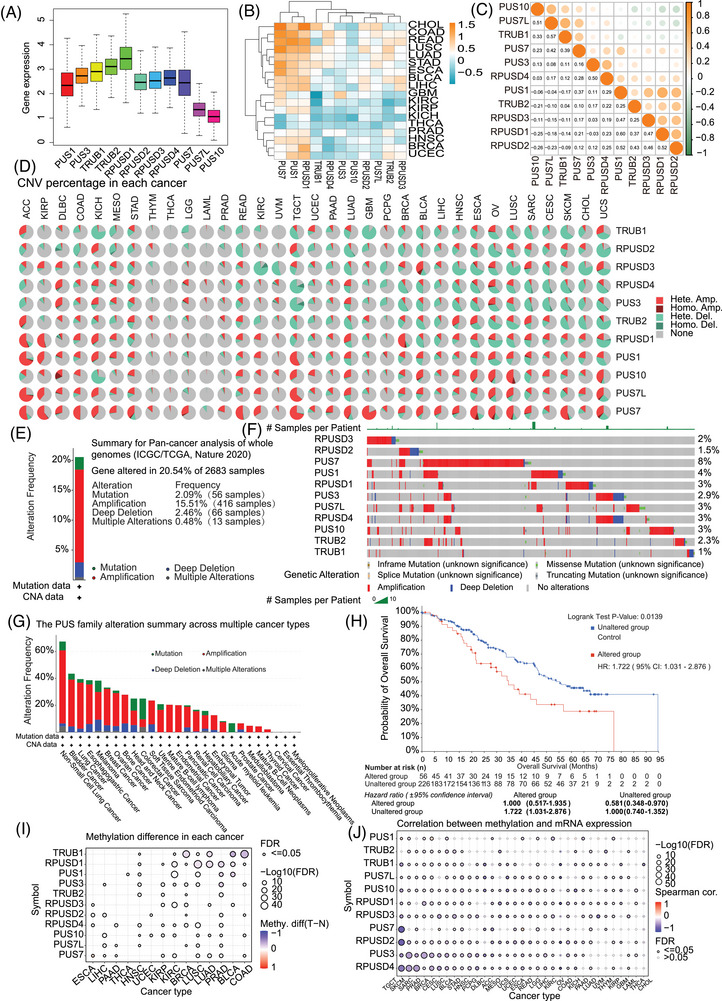
Pan‐cancer analysis of PUS family genes: Expression, genetic alterations, methylation, and multi‐platform characterization via cBioPortal. (A) Expression landscape of PUS family members across 33 TCGA cancer types (tumour samples only). (B) Heatmap displaying PUS family genes expression profiles in both cancer tissues and their corresponding noncancerous counterparts (differential expression analysis between tumour and adjacent normal tissues was restricted to cancer types with more than five paired normal samples in the TCGA database). (C) Correlation analysis conducted among PUS members. (D) The CNV information of the PUS family genes in pan‐cancer is summarized in the pie chart. (E) Overall occurrence rate of alterations in the PUS family genes across pan‐cancer. (F) Variations in PUS family gene levels across diverse cancer types. (G) Visual representation of genomic alterations related to the PUS family. (H) Kaplan–Meier analysis of overall survival (OS) comparing patients with PUS family gene alterations (PUS‐altered) and those without alterations (PUS‐unaltered) based on an integrated TCGA and ICGC dataset (*n* = 2683; hazard ratio [HR] = 1.722; log‐rank *p* = .0139). (I) DNA methylation patterns in tumour and normal samples of 13 cancer types. The bubble size represents the false discovery rate (FDR), and the bubble colour represents the fold‐change. The increased or decreased methylation is presented with red dots or blue dots respectively. A deeper colour indicates a larger difference. (J) The relationship of methylation with mRNA levels of PUS family genes. The darker the colour, the stronger is the correlation. *p < .05, **p < .01, ***p < .001.

The Graphical Abstract systematically illustrates the overall research framework of this study (Figure 1). First, we comprehensively analyzed the expression patterns of 11 PUS family members across multiple cancer types, based on transcriptomic data from 33 cancer types in the TCGA database. RPUSD1 exhibited markedly elevated expression across pan‐cancer tissues, followed by TRUB2, TRUB1, and PUS3. In contrast, PUS7L and PUS10 displayed lower expression levels (Figure 2A; Figure S1). We compared PUS expression levels between tumour tissues and adjacent normal tissues across 18 cancer types. Most PUS family members exhibited significantly elevated expression in adjacent normal tissues across pan‐cancer samples (Figure 2B; Figure S2).

In addition, the correlation among PUS family members were investigated (Figure 2C). A total of 36 gene pairs exhibited significant positive correlations, and 12 gene pairs showed significant negative correlations. Notably, PUS7 demonstrated significant positive correlations with all other family members (Figure 2C).

We analyzed OS, DSS, and PFS across 33 cancer types (Figure S3). Notably, elevated PUS7 expression consistently emerged as a significant adverse prognostic factor in multiple cancer types, strongly correlating with poorer clinical outcomes.

CNV analysis across 33 cancer types showed that heterozygous amplification and deletion were the most prevalent alterations (Figure 2D), with gene expression generally correlating positively with CNV events. SNV analysis with the GSCALite platform^28^ revealed frequent deleterious mutations in PUS7L and PUS7, particularly in uterine corpus endometrial carcinoma (UCEC) (Figure S4A). Notably, RPUSD4 expression showed significant positive correlation with CNV in 28 cancer types (Figure S4B), and mRNA levels were typically elevated in samples with amplifications but reduced in those with deletions (Figure S4C,D).

We further assessed genetic alterations in 2683 pan‐cancer samples from the ICGC/TCGA dataset with cBioPortal. Overall, 26.83% of samples exhibited PUS gene alterations, with CNVs (17.97%) occurring far more frequently than mutations (2.09%) (Figure 2E). Amplifications were the dominant CNV subtype (15.51%), especially in PUS7. Alteration frequencies ranged from 1% to 8% across cancer types (Figure 2F; Figure S5). Mutation‐dominant profiles were observed in acute myeloid leukaemia, while colorectal cancer showed the highest mutation frequency. Amplifications predominated in several cancers, including lung and bladder cancers (Figure 2G). Kaplan–Meier analysis revealed that PUS gene alterations were associated with significantly worse overall survival (HR = 1.722, *p* = .0139; Figure 2H).

We systematically analyzed the methylation profiles of PUS family genes across 33 cancer types and detected differential methylation in 14 cancer types (Figure 2I). In line with the known inverse relationship between methylation and gene expression,^35^ we observed negative correlations between methylation and mRNA levels for most PUS genes, with PUS7 and RPUSD2 showing the strongest associations in TGCT (Figure 2J).

### 3.2 Establishment of PUS score and the survival analysis according to the PUS score

**FIGURE 3:**
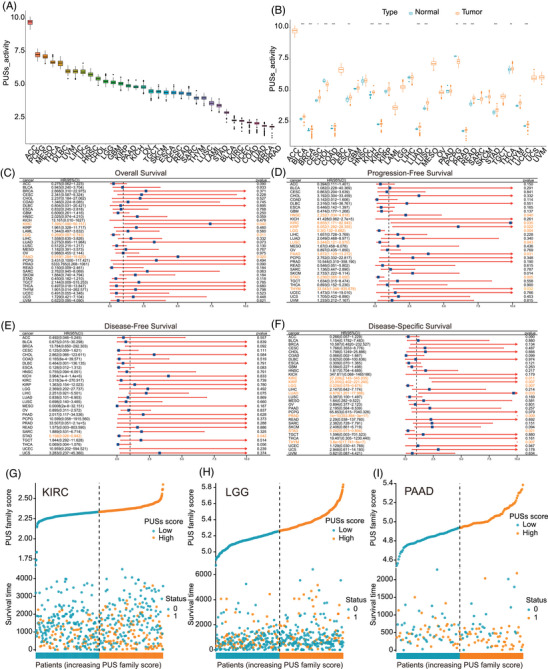
Establishing a PUS scoring system across cancers and survival analysis of the PUS score. (A) Rank‐order plot of PUS scores from lowest to highest across cancers. (B) The difference analyses of PUS score in tumour tissue samples versus paired adjacent normal tissue samples in indicated cancer types. (C–F) Forest plots of the Cox analysis results of the SKA score across cancers. (C) OS, (D) PFS, (E)DFS and (F) DSS. (G–I) Scatter plot of the distribution of OS with PUSs in KIRC, LGG and PAAD.

We constructed a scoring system based on the ssGSEA algorithm to quantify PUS family patterns across TCGA cancer types, referred to as the PUSs. Among the various cancer types, ACC exhibited the highest PUSs, while PRAD showed the lowest (Figure 3A). Compared with adjacent normal tissues, PUSs was significantly elevated in tumours, including BLCA, BRCA, CESC, COAD, ESCA, KICH, KIRC, KIRP, LUAD, LUSC, PRAD, READ, STAD, THCA, and UCEC, whereas it was decreased in CHOL and PCPG (Figure 3B). Univariate Cox regression analyses were performed for OS, DSS, disease‐free survival (DFS), and PFS. Higher PUSs was associated with increased risk in KIRC and PAAD, but acted as a protective factor in LGG in OS (Figure 3C). While PUSs served as a risk factor in HNSC, KIRC, KIRP, PAAD, and THYM, it was protective in LGG, LUSC, and STAD in PFS (Figure 3D). For DFS, due to the limited sample size, positive associations were only observed in STAD, where PUSs acted as a protective factor (Figure 3E). As for DSS, PUSs was protective in KIRC, KIPC, LUAD, PRAD, and THYM, but served as a risk factor in LGG and STAD (Figure 3F). Collectively, PUSs demonstrated prognostic predictive potential across multiple cancer types, with particularly strong predictive performance in KIRC, LGG, and PAAD (Figure 3G–I).

### 3.3 Association of PUSs with cancer stemness, immunogenicity, and hallmark pathway

**FIGURE 4:**
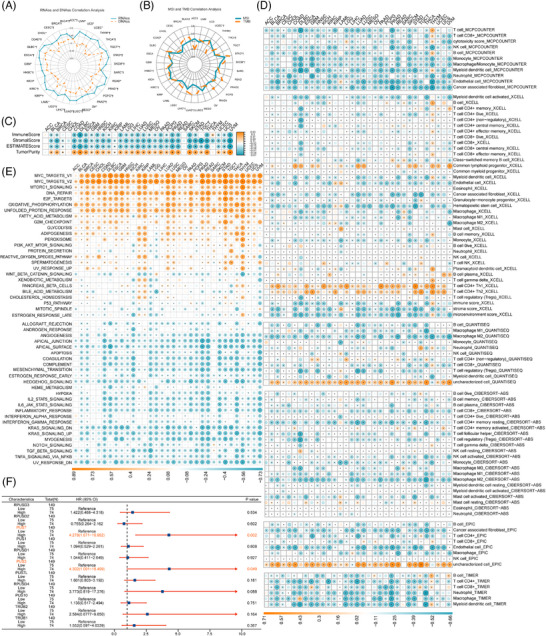
Association of PUSs with immunogenicity, cancer stemness and hallmark pathway. (A) Radar plot of the correlation between the PUS score and (red) DNAss or (blue) RNAss. (B) Radar plot of the correlation between the PUS score and (red) TMB or (blue) MSI. (C) Heatmap depicting the relationship of the PUS score with TME scores (including immune score, stromal score, ESTIMATE score and tumour purity). (D) A strong relationship was observed between multiple immune cells and the PUS score based on the TIMER 2.0 database. (E) Heatmap of the relationship of the PUS score with the 50 HALLMARK pathway score across 33 cancer types. (F) Forest plots of the unicox analysis results of the PUS family gene expression in PAAD in the TCGA cancer. *p < .05, **p < .01, ***p < .001.

Given the role of cancer stem cells in tumour progression and therapy resistance,^36^ we evaluated the relationship between PUSs and tumour stemness using RNA stemness score (RNAss) and DNA stemness score (DNAss). PUSs was positively correlated with RNAss across all cancers and with DNAss in most cancer types (Figure 4A), indicating a potential role in promoting stemness and tumour dedifferentiation.

MSI and TMB are established biomarkers for predicting responses to ICIs.^37^, ^38^, ^39^ We analyzed the correlations between PUSs and these biomarkers across cancer types (Figures S6 and S7). PUSs showed positive correlations with TMB in 18 cancers and with MSI in eight cancers, with the strongest associations observed in STAD (Figure 4B).

To investigate immune infiltration of the PUS7 family in cancer, we analyzed associations between PUSs and the TME. PUSs was negatively correlated with stromal, immune, and ESTIMATE scores in most cancers, and positively associated with tumour purity (Figure 4C). Additionally, PUSs was inversely correlated with the expression of most immune checkpoint genes (Figure S8) and with immune cell infiltration across multiple algorithms (Figure 4D), indicating a link to an immunosuppressive TME.

We performed GSVA on 50 hallmark pathways. PUSs was strongly associated with proliferation and cell cycle‐related pathways, including E2F targets, G2M checkpoint, MYC targets, mitotic spindle, and DNA repair (Figure 4E). Additionally, PUSs was positively linked to metabolic pathways such as fatty acid metabolism, glycolysis, and oxidative phosphorylation, suggesting elevated metabolic activity in PUS‐high tumours.

Given the strong prognostic relevance of PUSs observed in KIRC, LGG, and PAAD, we further investigated the expression patterns and functional significance of PUS family genes specifically in the TCGA‐PAAD cohort. As shown in Figure S8A, the expression levels of PUS1, RPUSD1, and RPUSD3 were positively correlated with RNAss and DNAss but negatively correlated with stromal score and ESTIMATE score. The results suggest that PUS family members, including PUS1, RPUSD1, RPUSD3, and PUS7, may contribute to both enhanced tumour stemness and the establishment of an immunosuppressive microenvironment in PAAD. Furthermore, univariate and multivariate Cox regression analyses confirmed that PUS7 serves as an independent adverse prognostic factor for PAAD patients (HR = 3.824, 95% CI: 1.475–9.916, *p* = .006; Table 1; Figure 4F).

**TABLE 1:** Unicox and multicox analysis of PUS family genes in PAAD.

		Univariate analysis		Multivariate analysis	
Gene	Cases	Hazard ratio (95% CI)	*p‐*value	Hazard ratio (95% CI)	*p‐*value
RPUSD3	150	1.442 (.469–4.318)	.534		
RPUSD2	150	.756 (.264–2.163)	.602		
PUS7	150	4.279 (1.672–10.953)	**.002**	3.824 (1.475–9.916)	**.006**
PUS1	150	1.094 (.529–2.261)	.808		
RPUSD1	150	1.044 (.412–2.649)	.927		
PUS3	150	4.303 (1.001–18.499)	.050	3.262 (.710–14.987)	.129
PUS7L	150	1.601 (.803–3.192)	.181		
RPUSD4	150	3.773 (.819–17.376)	.088		
PUS10	150	1.136 (.517–2.494)	.751		
TRUB2	150	2.584 (.678–9.850)	.164		
TRUB1	150	1.552 (.597–4.034)	.367		

To explore the potential relationship between PUS7 expression and drug sensitivity, we performed a correlation analysis using the CellMiner database. The results demonstrated that high PUS7 expression was positively associated with increased sensitivity to several drugs, including Raloxifene, Bafetinib, Dimethylaminoparthenolide and Nilotinib, suggesting these agents may have greater therapeutic efficacy in PAAD patients with elevated PUS7 expression. Conversely, PUS7 expression was negatively correlated with sensitivity to Dasatinib and Staurosporine, implying that patients with high PUS7 expression may respond poorly to these agents and require careful therapeutic consideration. The top 15 representative drugs with the most significant correlations to PUS7 expression are presented in Figure S8B.

Together, these findings highlight the PUS family as potential regulators of higher stemness and immunosuppressive TME, and PUS7 exhibits great potential in PDAC.

### 3.4 Elevated PUS7 expression is significantly associated with poor prognosis in PDAC patients

**FIGURE 5:**
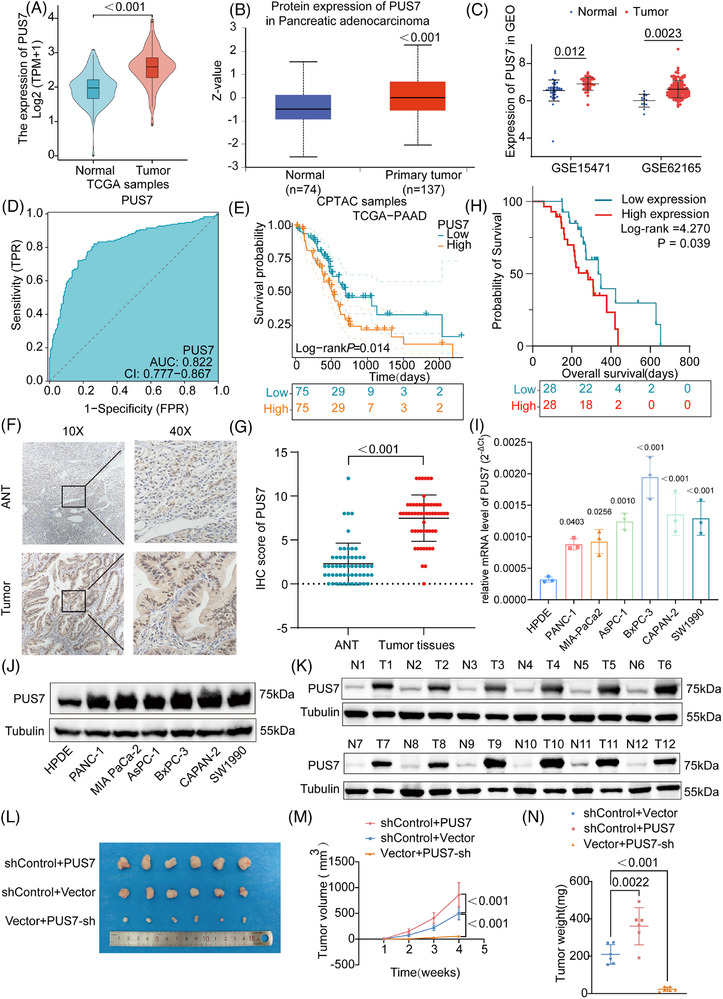
Elevated PUS7 expression is significantly associated with poor prognosis in PDAC patients. (A) Analysis of RNA‐seq data from the TCGA database revealed significantly increased PUS7 mRNA expression in PDAC tissues compared with adjacent non‐tumourous pancreatic tissues. Statistical significance was determined using the Wilcoxon rank‐sum test. (B) PUS7 protein expression levels in PDAC and matched normal tissues were analyzed using data from the CPTAC database. Statistical significance was determined using the Wilcoxon rank‐sum test. (C) Expression levels of PUS7 mRNA in PDAC were further validated across multiple GEO datasets. Statistical significance was determined using the Wilcoxon rank‐sum test. (D) Diagnostic performance of PUS7 was evaluated by receiver operating characteristic (ROC) curve analysis using combined data from the TCGA and GTEx databases. ROC curve analysis was performed, and the area under the curve (AUC) was calculated to evaluate diagnostic performance. (E) Kaplan–Meier survival analysis of TCGA PDAC patients stratified by PUS7 expression levels. Statistical significance was determined using the log‐rank test. (F) IHC staining images showing PUS7 expression in paired tumour and adjacent non‐tumourous pancreatic tissues (ANT), scale bar = 200 µm, magnifications, 10× and 40×. (G) Quantification of IHC scores demonstrates significantly higher PUS7 expression in tumour tissues (*n* = 56 paired samples, paired two‐tailed Student's *t*‐test). The H‐score evaluation was performed by two independent pathologists and showed excellent inter‐observer agreement (Cohen's κ = 0.82). (H) Overall survival analysis of 56 clinical PDAC patients based on PUS7 expression levels. Statistical significance was determined using the log‐rank test. (I) Relative mRNA expression levels of PUS7 in PDAC cell lines (PANC‐1, MIA PaCa‐2, ASPC‐1, BXPC‐3, SW1990, CAPAN‐2) and normal pancreatic ductal epithelial cells (HPDE), determined by qRT‐PCR (*n* = 3 independent experiments, one‐way ANOVA followed by Tukey's multiple comparisons test). (J) Western blot analysis of PUS7 protein levels in PDAC cell lines and HPDE. (K) Western blot analysis of PUS7 expression in 12 paired clinical PDAC and adjacent normal pancreatic tissue samples. (L) Representative tumour images from subcutaneous xenografts in nude mice injected with control, PUS7‐overexpressing, or PUS7‐silenced PDAC cells. (M) Tumour growth curves showing the longitudinal measurement of tumour volumes from the experiment in (L) (*n* = 6 biological replicates of mice per group, two‐way repeated measures ANOVA followed by Tukey's multiple comparisons test). (N) Quantification of tumour weights at the endpoint (*n* = 6 biological replicates of mice, one‐way ANOVA followed by Tukey's multiple comparisons test). Data are presented as mean ± SEM.

To further elucidate the relationship between PUS family gene expression and tumour behaviour, we validated the mRNA and protein levels of PUS7 in PDAC. Integrated analyses of TCGA, CPTAC, and GSE datasets consistently revealed a significant upregulation of PUS7 in PDAC tissues (Figure 5A–C). Receiver operating characteristic curve based on combined TCGA and GTEx datasets yielded an area under the curve (AUC) of .822 (95% CI: .777–.867), highlighting the strong diagnostic potential of PUS7 in PDAC (Figure 5D). Kaplan–Meier survival analysis further demonstrated that higher PUS7 expression was significantly associated with poorer overall survival in PDAC patients (Figure 5E).

To validate these findings in clinical specimens, we analyzed tumour tissues from a cohort of 56 PDAC patients. IHC staining confirmed significantly higher PUS7 expression in tumour tissues compared with adjacent non‐tumourous pancreatic tissues (ANT) (Figure 5F,G). Notably, elevated PUS7 expression correlated with reduced overall survival, reinforcing its potential as a negative prognostic marker (Figure 5H). We validated the PUS7 mRNA level and protein level in multiple PDAC cell lines relative to normal pancreatic ductal epithelial cells (Figure 5I,J). The complete raw Ct value distribution underlying these analyses is shown in Figure S9K to ensure transparency and reproducibility. Western blot analysis of paired tumour and adjacent normal tissues from 12 PDAC patients further confirmed significantly increased PUS7 protein levels in tumour samples (Figure 5K). Collectively, these findings showed that PUS7 was upregulated in PDAC and strongly associated with poor prognosis, underscoring its potential as a biomarker and therapeutic target.

We established PANC‐1 and MIA PaCa‐2 cell lines with stable PUS7 overexpression or knockdown, and confirmed the alterations in PUS7 expression by western blotting (Figures S9A,B and S10A,B). Functional assays, including wound healing, Transwell migration, and invasion, colony formation and EdU incorporation, demonstrated that PUS7 knockdown significantly suppressed the migratory, invasive, clonogenic, and proliferative capacities of PDAC cells (Figure S9C–J). Conversely, PUS7 overexpression enhanced these malignant phenotypes (Figure S10C–J). We next conducted subcutaneous xenograft experiments in nude mice. PUS7‐depleted cells exhibited significantly reduced volume and weight compared with controls, while PUS7‐overexpressing tumours showed markedly accelerated growth (Figure 5L–N). Together, these results showed that PUS7 silencing inhibits and its overexpression promotes tumour growth both in vitro and in vivo.

### 3.5 PUS7 promotes the formation of NETs in pancreatic cancer

**FIGURE 6:**
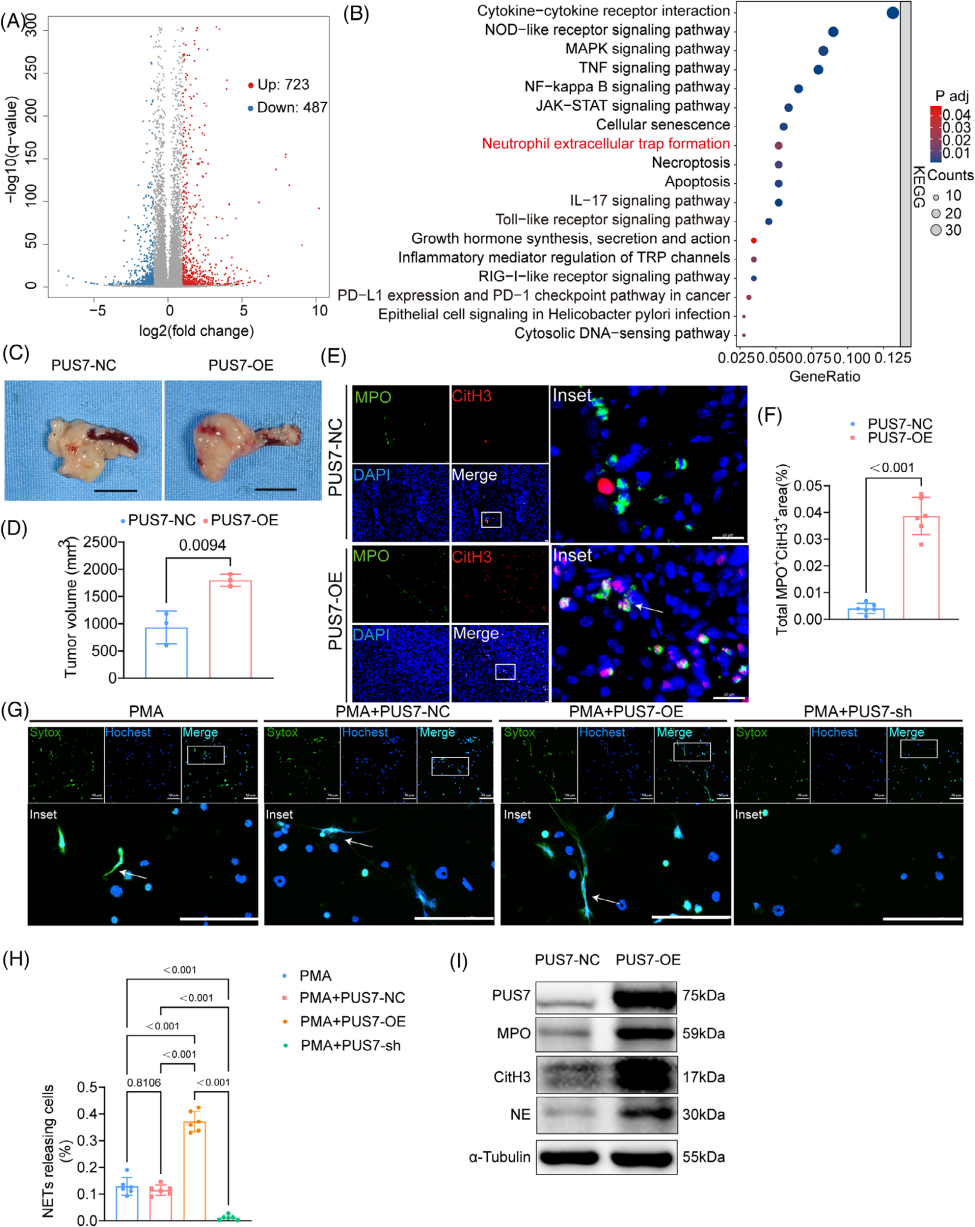
PUS7 promotes the formation of NETs in pancreatic cancer. (A) Volcano plot illustrating DEGs between PUS7‐OE and PUS7‐NC PANC‐1 cells. Red and blue dots represent significantly upregulated and downregulated genes, respectively. (B) KEGG pathway enrichment analysis of DEGs identified in PUS7‐OE cells (*p* < .05). (C) Measurement of orthotopic pancreatic tumour size in PUS7‐NC and PUS7‐OE mice. (D) Quantification of tumour weights from panel (C) (*n* = 3 biological replicates of mice, unpaired *t*‐test). (E) Representative immunofluorescence staining images of NETs markers in pancreatic tumour tissues, scale bar = 200 µm. (F) Quantification of the data presented in panel (*n* = 3 biological replicates of mice per group, Unpaired *t*‐test) (E). (G) Representative fluorescence micrographs. Blue: Hoechst 33342 (all nuclei); Green: SYTOX Green (NETs). White arrows indicate NETosis‐positive cells. Scale bar: 20 µm. (H) Quantitative analysis of the percentage of NETosis‐positive cells. (*n* = 3 independent experiments, One‐way ANOVA followed by Tukey's multiple comparisons test). (I) Western blot analysis of NETs‐related proteins (MPO, NE, CitH3) in tumours from PUS7‐NC and PUS7‐OE mice.

To further investigate the regulatory mechanisms underlying PUS7 function in PDAC, we performed bulk RNA sequencing on PUS7 overexpression PANC‐1 cells. Differential expression analysis identified 1210 differentially expressed genes (DEGs), including 723 upregulated and 487 downregulated genes (Figure 6A). GO analysis revealed that the DEGs were primarily enriched in biological processes related to immune system regulation, cell proliferation, and cell adhesion. In terms of molecular function, enrichment was observed in chemokine, cytokine, and receptor binding activities (Figure S11A). KEGG pathway analysis demonstrated that DEGs were significantly enriched in multiple immune and inflammation‐related pathways, including cytokine–cytokine receptor interaction, neutrophil extracellular trap formation, NOD‐like receptor signalling and IL‐17 signalling (Figure 6B). RNA sequencing analysis of tumour cells revealed that PUS7 overexpression significantly altered the expression of multiple cytokines and chemokines implicated in neutrophil recruitment and activation. Notably, several soluble factors associated with NET induction, including CXCL2, CXCL8, and IL‐6, were markedly upregulated in PUS7‐overexpressing tumour cells compared with controls (Figure S11B). Further GSEA analysis showed that PUS7 overexpression was positively associated with various immune‐related pathways, including B cell receptor signalling, IL‐17 signalling, PD‐L1 and PD‐1 checkpoint pathways, TH1 and TH2 cell differentiation, Toll‐like receptor signalling, JAK‐STAT signalling, NF‐κB signalling, and NETs formation (Figure S11C).

We adopted a hypothesis‐driven approach to investigate whether PUS7 contributes to PDAC progression through modulation of NETs formation. To this end, we established a hydrodynamic mouse model of pancreatic cancer. PUS7 overexpression markedly increased tumour burden compared with controls (Figure 6C,D). Throughout the experimental period, mice in the PUS7‐OE group exhibited earlier and more significant body weight loss compared with the control group, which is consistent with the higher terminal tumour burden in this group, indicating that body weight changes effectively reflect the systemic impact of tumour progression (Figure S11D). Moreover, PUS7 bidirectionally regulates NETs formation. This was evidenced in vivo by immunofluorescence, which showed upregulated levels of key NETs markers (MPO and CitH3) in PUS7‐overexpressing tumours (Figure 6E,F). In vitro, PUS7 overexpression promoted NETs formation, whereas its knockdown reduced NETs, as quantified by Sytox Green staining and confirmed by western blot analysis (Figure 6G–I). Collectively, these findings suggest that PUS7 overexpression may promote NETs formation in PDAC, which may, in turn, contribute to enhanced tumour progression.

### 3.6 PUS7 reprograms macrophage infiltration and polarization in pancreatic cancer

**FIGURE 7:**
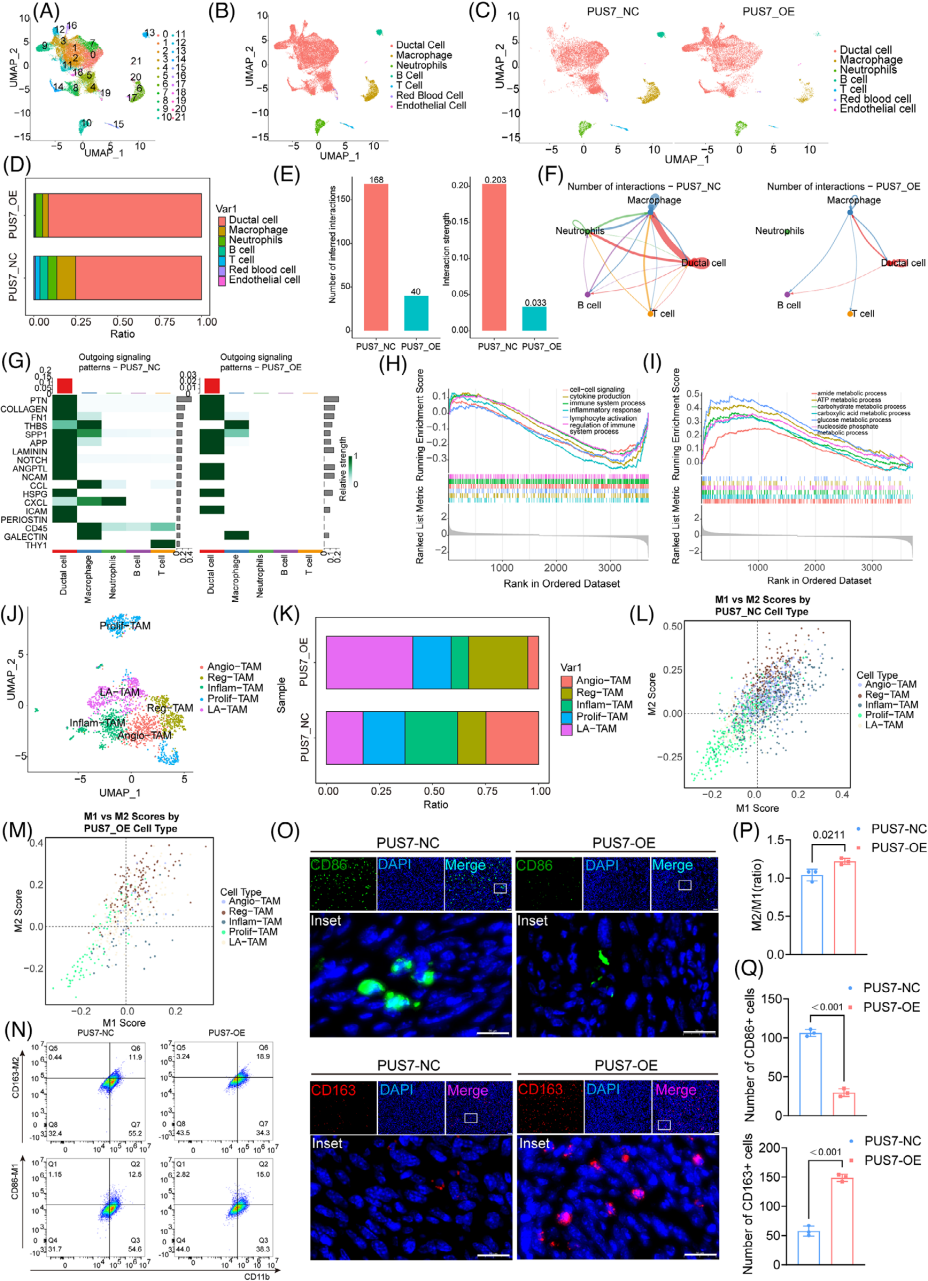
Single‐cell sequencing analysis reveals the regulatory impact of PUS7 on the tumour immune microenvironment in pancreatic cancer. (A) UMAP plots of two pancreatic cancer single‐cell datasets (derived from tumour tissues of *n* = 1 mouse per group), showing 22 distinct cell clusters identified through dimensionality reduction. (B) Annotation based on marker gene expression categorized the cells into seven major cell types: ductal cells, macrophages, neutrophils, B cells, T cells, endothelial cells, and red blood cells. (C) UMAP plots of cells grouped by PUS7 expression levels: PUS7 control group (left) and PUS7 overexpression group (right). (D) Proportions of each cell type in the PUS7 control and PUS7 overexpression groups. (E) Quantitative analysis of intercellular interactions ranked by frequency and strength. (F) Network diagram illustrating how PUS7 affects intercellular interactions; the thicker the connecting lines, the stronger the interactions between cells. (G) Heatmap showing the signalling pathway intensities of cell‐cell interactions in the PUS7 control group (left) and PUS7 overexpression group (right), highlighting pathways such as NOTCH, PERIOSTIN, SPP1, CCL, CD45, CXCL, and THY1. (H, I) GSEA results depicting the impact of PUS7 on immune‐related pathways (H) and metabolism‐related pathways (I) in TCGA‐PAAD datasets. (J) Annotation of macrophages into five subtypes based on marker gene expression: angiogenic TAMs (Angio‐TAMs), immunoregulatory TAMs (Reg‐TAMs), inflammatory cytokine‐enriched TAMs (Inflam‐TAMs), proliferative TAMs (Prolif‐TAMs), and lipid‐associated TAMs (LA‐TAMs). (K) Proportions of each macrophage subtype in the PUS7 control and PUS7 overexpression groups. (L, M) Dot plots illustrating the correlation of each macrophage subtype with M1 (L) and M2 (M) polarization states in the PUS7 control and overexpression groups. (N) Representative flow cytometry pseudocolour plots showing macrophage polarization status in tumours following PUS7 overexpression. (O) Immunofluorescence staining of CD86^+^ (M1 macrophage marker) and CD163^+^ (M2 macrophage marker) cells in an orthotopic pancreatic cancer mouse model 200× magnification), scale bar = 20 µm. (P) Statistical analysis of the M2/M1 macrophage ratio based on data from panel (N). Data are presented as mean ± SEM for each group (*n* = 3 independent experiments, unpaired *t*‐test). (Q) Quantification of the percentages of CD86^+^ and CD163^+^ cells from panel (O). Data are presented as mean ± SEM for each group (*n* = 3 biological replicates of mice per group, unpaired *t*‐test).

We performed scRNA‐seq on orthotopic pancreatic tumours. A total of 27 230 high‐quality cells were retained for downstream analysis. We identified 22 distinct cellular clusters (Figure 7A), with marker gene expression profiles used for cell‐type annotation (Figure S12A). Seven major cell types were identified: ductal epithelial cells, macrophages, neutrophils, B cells, T cells, endothelial cells, and erythrocytes (Figure 7B,C; Figure S12B). The cell identities were confirmed by canonical markers: *Col3a1* and *Col1a1* for ductal epithelial cells; *C1qc* and *C1qa* for macrophages; *S100a8* and *S100a9* for neutrophils; *Cd79a* and *Cd79b* for B cells; *Cd3e*, *Cd3d*, and *Cd3g* for T cells; *Cldn5* and *Cdh5* for endothelial cells; and *Hbb‐bt* for erythrocytes (Figure S12C,D).

PUS7 overexpression led to a marked increase in ductal epithelial cells and a concomitant reduction in immune cell populations, including macrophages, B cells, and T cells (Figure 7D). Cell chat analysis reveals that, in the PUS7‐OE group, both the number and strength of interactions among cell types were significantly reduced (Figure 7E), with the most notable decline observed in neutrophil‐immune cell crosstalk (Figure 7F). Further analysis revealed a downregulation of intercellular signalling from parenchymal cells, particularly pathways involving NOTCH, PERIOSTIN, SPP1, CCL, CD45, CXCL, and THY1, which mediate communication between parenchymal and immune cells (Figure 7G). KEGG‐pathway‐enrichment analysis of ductal epithelial cells showed decreased enrichment of immune activation pathways in the PUS7‐OE group (Figure 7H), alongside enhanced enrichment of metabolic pathways such as glucose and ATP metabolism (Figure 7I).

To dissect the impact of PUS7 on macrophage dynamics, we clustered the macrophage population (Figure S12E) and annotated several functionally distinct TAM subtypes,^40^ including angiogenic TAMs (Angio‐TAMs), regulatory TAMs (Reg‐TAMs), inflammatory TAMs (Inflam‐TAMs), proliferative TAMs (Prolif‐TAMs), and lipid‐associated TAMs (LA‐TAMs) (Figure 7J; Figure S12F,G). PUS7 overexpression resulted in a substantial reduction of antitumour Inflam‐TAMs, while LA‐TAMs, Angio‐TAMs and Reg‐TAMs were significantly expanded (Figure 7K–M; Figure S12H,I), suggesting that PUS7 influences TAM towards an immune‐suppressed phenotype.

To validate these findings, we co‐culture THP‐1‐derived M0 macrophages with PUS7 conditioned medium. Flow cytometry analysis indicated that, compared with controls, PUS7 overexpression significantly increased the M2/M1 macrophage ratio (Figure 7N,P), whereas PUS7 knockdown significantly decreased the M2/M1 ratio (Figure S11F,G). Immunofluorescence staining of pancreatic tissues further supported this observation, showing decreased numbers of CD86 and increased CD206 macrophages in the PUS7‐OE group (Figure 7O,Q).

Together, these results demonstrate that PUS7 overexpression impairs the antitumour function of macrophages by skewing their differentiation towards an immunosuppressive phenotype.

### 3.7 PUS7 regulates macrophage polarization through modulation of NETs

**FIGURE 8:**
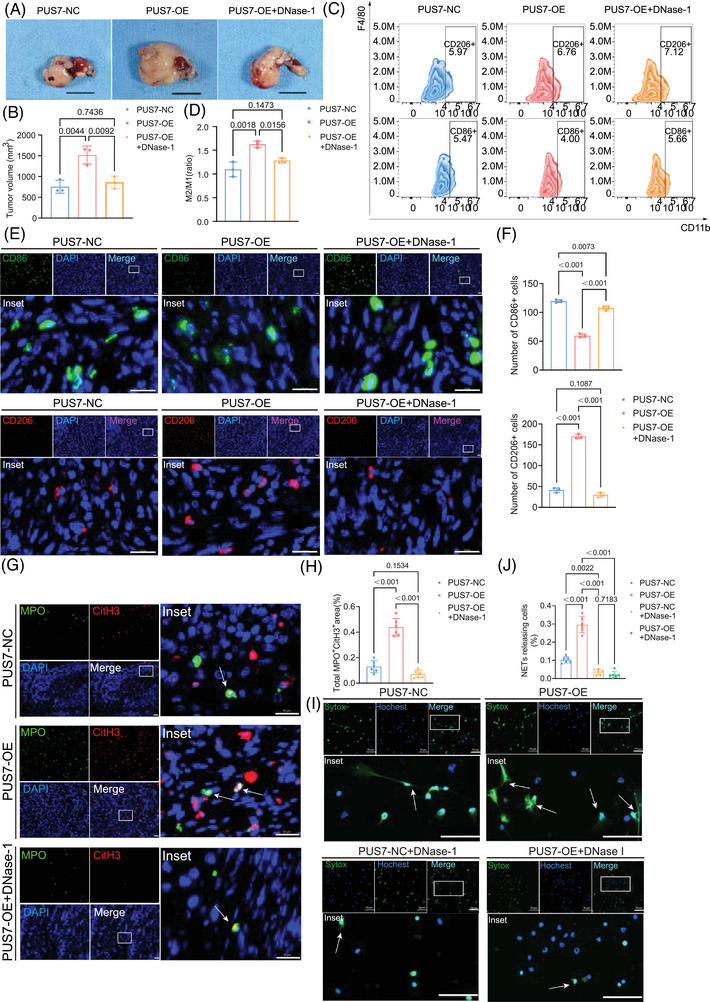
PUS7 regulates macrophage polarization through modulation of NETs. (A) Measurement of orthotopic pancreatic tumour size in control, PUS7‐overexpressing, and PUS7‐overexpressing mice treated with the NETs inhibitor DNase‐1. (B) Quantification of tumour weights from panel (A) (*n* = 3 mice per group, one‐way ANOVA followed by Tukey's multiple comparisons test.). (C) Flow cytometry analysis of CD86^+^ (M1 macrophage marker) and CD206^+^ (M2 macrophage marker) cells in tumours from the three groups. (D) Analysis of the M2/M1 macrophage ratio in each group (*n* = 3 independent experiments, One‐way ANOVA followed by Tukey's multiple comparisons test). (E) Representative immunofluorescence staining of CD86^+^ and CD206^+^ cells in orthotopic pancreatic tumours. Scale bar, 20 µm. (F) Quantification of CD86^+^ and CD206^+^ cells as a percentage of total cells in each group (*n* = 3 independent experiments, One‐way ANOVA followed by Tukey's multiple comparisons test). (G) Representative immunofluorescence images of tumour sections stained for the NETs markers CitH3 (red) and MPO (green). Nuclei are counterstained with DAPI (blue). Scale bar, 20 µm. (H) Quantification of the MPO^+^CitH3^+^ area per field (*n* = 3 biological replicates of mice, one‐way ANOVA followed by Tukey's multiple comparisons test). (I) Representative fluorescence micrographs. Blue: Hoechst 33342 (all nuclei); Green: SYTOX Green (NETs). White arrows indicate NETosis‐positive cells. Scale bar: 20 µm. (J) Quantitative analysis of the percentage of NETosis‐positive cells (*n* = 3 independent experiments, One‐way ANOVA followed by Tukey's multiple comparisons test). Data represent mean ± SEM from at least three independent experiments.

To determine whether PUS7 promotes PDAC progression through NETs, we established an orthotopic tumour model by implanting Pan02 cells stably overexpressing PUS7 (Pan02‐PUS7) into C57BL/6 mice. Treatment with DNase I, an enzyme that degrades extracellular DNA, completely abolished the tumour‐promoting effect of PUS7 overexpression, indicating that NETs clearance alleviates PUS7‐driven tumour growth (Figure 8A,B). Longitudinal monitoring of body weight revealed that PUS7‐overexpressing mice exhibited earlier and greater weight loss, which was prevented by DNase I, further supporting the systemic impact of NETs (Figure S11E). Flow cytometry and immunofluorescence analysis of tumour tissues further revealed that DNase I administration significantly reduced M2 macrophages while increasing M1 macrophages in PUS7‐overexpressing tumours (Figure 8C–F). Moreover, tumours from DNase I‐treated PUS7‐overexpressing mice exhibited decreased levels of the NETs markers MPO and CitH3, confirming that DNase I suppresses PUS7‐induced NETs formation in vivo (Figure 8G,H).

To directly assess the impact of PUS7 on NETosis, we performed an in vitro assay. Neutrophils were treated with conditioned medium from Pan02‐PUS7 cells. Consistent with the in vivo findings, this treatment significantly increased the percentage of neutrophils undergoing NETosis, an effect that was abolished by the addition of DNase I (Figure 8I,J). This result provides direct evidence that PUS7 overexpression enhances NETosis in a cell‐extrinsic manner. However, whether NETs directly mediate PUS7‐induced macrophage polarization remained unclear.

To rigorously validate the specificity of the in vivo effect, we performed parallel DNase I treatment in mice bearing control tumours. In contrast to its potent effects in the PUS7‐overexpressing setting, DNase I treatment did not significantly alter tumour volume (Figure S13A,B) or macrophage polarization status (Figure S13C–F) in Vector control tumours, nor did it cause significant body weight loss (Figure S13G). This direct comparison demonstrates that the antitumour and immunomodulatory effects of NETs degradation are strictly dependent on the PUS7‐overexpressing state.

To directly determine whether NETs function as the essential intermediate linking PUS7‐overexpressing tumour cells to macrophage phenotypic reprogramming, we established an in vitro Transwell‐based sequential co‐culture system. Neutrophils were first stimulated with conditioned medium derived from Pan02‐PUS7 cells to induce NET formation. The resulting NETs‐containing supernatant was subsequently collected and applied to macrophages. To specifically assess the contribution of NETs, DNase I (1.5 U/mL) was added after neutrophil stimulation to degrade extracellular DNA without affecting upstream neutrophil activation.

Strikingly, exposure to NETs‐containing supernatant derived from PUS7‐overexpressing tumour cells robustly induced M2‐like macrophage polarization, as evidenced by a significantly increased CD206/CD86 ratio. Importantly, this effect was markedly reversed upon DNase I treatment, demonstrating that degradation of NETs effectively abolished PUS7‐driven macrophage polarization (Figure S13H,I). These results provide direct mechanistic evidence that NETs are a necessary and non‐redundant mediator of PUS7‐induced macrophage phenotypic reprogramming.

To further validate the functional requirement of neutrophils and NETs in vivo, we next performed neutrophil depletion experiments using an anti‐Ly6G antibody. Effective depletion of neutrophils markedly attenuated the accelerated tumour growth induced by PUS7 overexpression (Figure S13J,K). Consistently, analysis of the tumour immune microenvironment revealed that neutrophil depletion significantly reduced the accumulation of M2‐polarized macrophages in PUS7‐overexpressing tumours, while partially restoring macrophage polarization towards a less immunosuppressive phenotype (Figure S13L–N).

These results indicate that neutrophils are required for PUS7‐mediated tumour growth and macrophage polarization in vivo.

RIP‐seq analysis comparing PUS7‐overexpressing (OE) and control (NC) cells revealed a set of differentially enriched mRNAs (Figure S14A). Functional enrichment showed that these PUS7‐bound transcripts are involved in PI3K–Akt, Ras, Hippo, Wnt, and HIF‐1 signalling, as well as immune‐related processes including T cell differentiation and activation (Figure S14B,C), suggesting that PUS7 promotes NETs formation and M2 macrophage polarization by selectively regulating RNAs that orchestrate tumour‐supportive immune signalling.

### 3.8 Identification of candidate small‐molecule inhibitors targeting PUS7 for PDAC treatment

**FIGURE 9:**
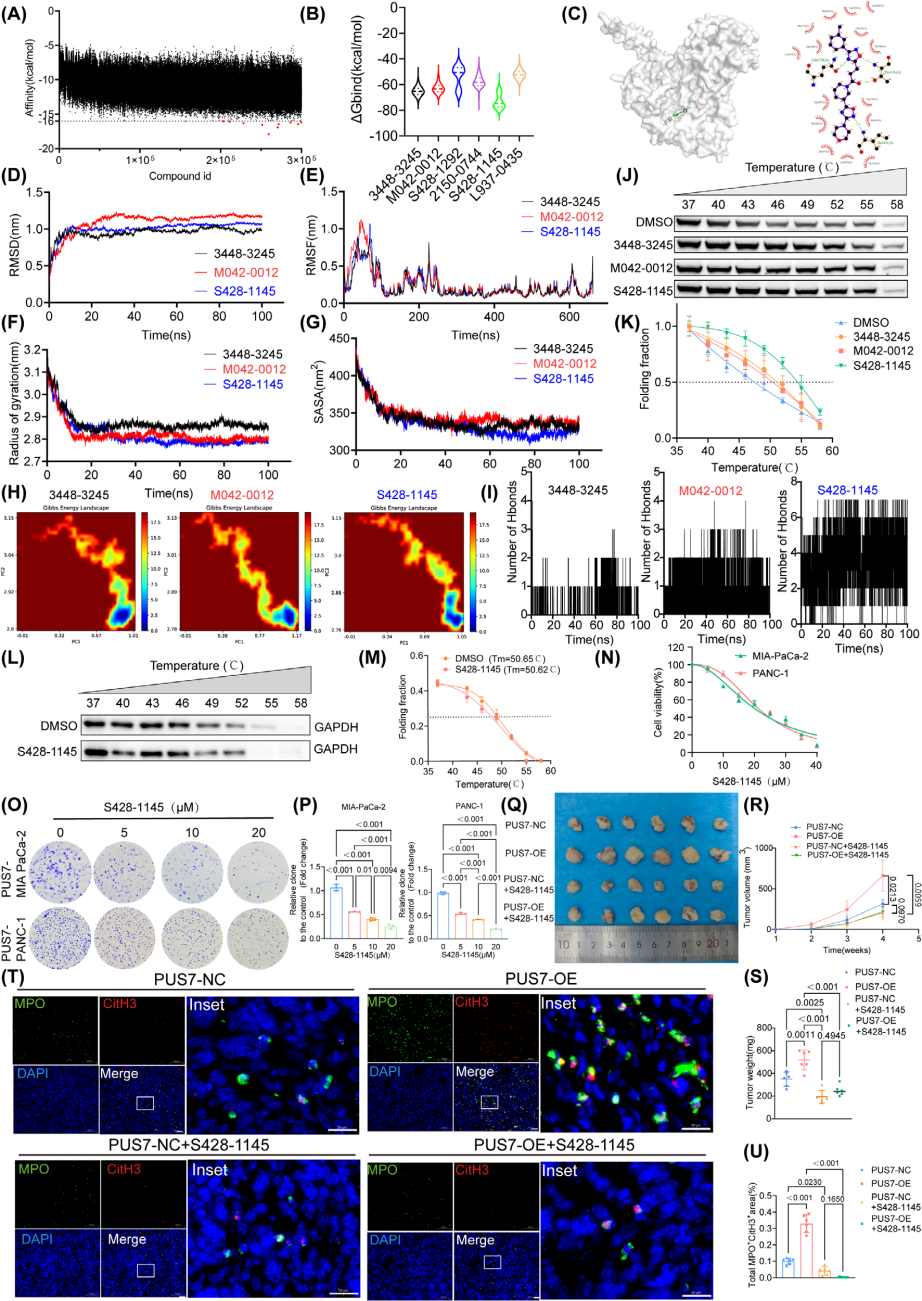
Identification of candidate small‐molecule inhibitors targeting PUS7 for PDAC treatment. (A) Binding affinity scores of approximately 300 000 small‐molecule compounds screened against PUS7. (B) Binding free energy (ΔG bind) values of PUS7 complexes with 3448‐3245, M042‐0012, and S428‐1145. (C) Molecular docking model of PUS7 in complex with S428‐1145.(D) Root mean square deviation (RMSD) trajectories of the three PUS7‐ligand complexes during molecular dynamics simulations. (E) Root mean square fluctuation (RMSF) analysis showing residue flexibility of PUS7 in complex with each compound. (F) Radius of gyration (Rg) profiles of the three PUS7‐ligand complexes. (G) Solvent‐accessible surface area (SASA) of the PUS7‐ligand complexes over simulation time. (H) Free energy landscape plots based on RMSD and Rg values; low‐energy conformational states are shown in blue (stable), while high‐energy states are shown in red (unstable). (I) Number of hydrogen bonds formed between PUS7 and each ligand during simulation. (J) CETSA showing increased thermal stability of PUS7 in cell lysates upon treatment with 3448‐3245, M042‐0012, and S428‐1145, with S428‐1145 exhibiting the most pronounced effect. (K) Nonlinear curve fitting of CETSA data from panel J for statistical analysis (mean ± SD, *n* = 3). (L) Representative immunoblots of soluble GAPDH from CETSA assays with S428‐1145 or DMSO treatment. (M) Quantification of GAPDH levels (mean ± SD, *n* = 3). The lack of effect confirms the specific stabilization of PUS7 by S428‐1145. (N) Dose‐response curves of S428‐1145 in MIA PaCa‐2 and PANC‐1 cells. Cells were treated with increasing concentrations of S428‐1145 for 72 h, and cell viability was measured using the CCK‐8 assay. IC_50_ values were calculated as indicated. (O, P) Clonogenic assays of MIA PaCa‐2 and PANC‐1 cells treated with 5, 10, or 20 µM S428‐1145 for 10–14 days. Representative images of colonies are shown (O), and quantitative analysis of colony numbers is presented (P). Data are presented as mean ± SEM from three independent experiments. (Q) Representative images of xenograft tumours. (R) Tumour growth curves (*n* = 6 mice per group, two‐way repeated measures ANOVA). (S) Bar graph of tumour weights at the endpoint. (T) Representative immunofluorescence images of tumour sections stained for the NETs markers CitH3 (red) and MPO (green). Nuclei are counterstained with DAPI (blue). Scale bar, 20 µm. (U) Quantification of the MPO^+^CitH3^+^ area per field (*n* = 3 biological replicates of mice, one‐way ANOVA with Tukey's multiple comparisons test).

To identify potential small‐molecule inhibitors targeting PUS7, we conducted virtual screening of approximately 300 000 compounds from Chemdiv using AutoDock‐GPU for molecular docking against the PUS7 protein structure (Figure 9A). Based on binding affinity scores, the top 6 candidate compounds were selected for further analysis. We conducted a 100 ns all‐atom molecular dynamics simulation, and molecular mechanics/generalized born surface area (MM/GBSA) were calculated (Figure 9B). Among which three—S428‐1145, 3448‐3245, and M042‐0012—exhibited the lowest binding free energies and were selected for evaluation. The structure of PUS7 and S428‐1145 was demonstrated in Figure 9C. The RMSD plots showed that all three complexes maintained stable conformations throughout the simulation period (Figure 9D). Notably, the PUS7‐M042‐0012 complex exhibited greater flexibility within the binding pocket compared with the others (Figure 9E). The PUS7‐S428‐1145 complex displayed the lowest Rg value, suggesting a more compact and stable binding conformation (Figure 9F). SASA gradually decreases with the simulation time (Figure 9G). The Gibbs free energy landscape revealed that all complexes showed relative stable state during simulation. (Figure 9H). Notably, S428‐1145 established the highest number of persistent hydrogen bonds, implying tighter and more specific binding within the active site (Figure 9I). To validate ligand binding in vitro, we performed a CETSA. Treatment with S428‐1145 specifically increased the thermal stability of PUS7 compared with other compounds (Figure 9J,K), and this effect was not observed for the unrelated control protein GAPDH (Figure 9L,M). Together, these data indicate a direct and specific interaction between S428‐1145 and PUS7, supporting its potential as a targeted small‐molecule inhibitor.

To systematically evaluate the druggability potential of S428‐1145, we conducted comprehensive in vitro and in vivo validation. First, we determined the half‐maximal inhibitory concentration (IC_50_) of S428‐1145 in two human PDAC cell lines. The IC_50_ values were 20 µM in MIA PaCa‐2 cells and 20.68 µM in PANC‐1 cells, respectively (Figure 9N). Based on these results, concentration gradients of 5, 10, and 20 µM were selected for subsequent functional validation in PUS7‐high PDAC cells. Treatment with S428‐1145 dose‐dependently and significantly inhibited the clonogenic ability of tumour cells (Figure 9O,P), confirming its anti‐proliferative activity in vitro. More importantly, further in vivo experiments demonstrated that S428‐1145 (25 mg/kg, once daily) significantly inhibited tumour growth (Figure 9Q–S) and downregulated key NETs biomarkers such as Cit‐H3 and MPO in tumour tissues (Figure 9T,U).

## 4 DISCUSSION

RNA modifications have been established as critical epigenetic regulators in cancer, contributing to the modulation of gene expression and cellular plasticity. Among these, pseudouridylation (Ψ) represents one of the most abundant and evolutionarily conserved RNA modifications, known to influence RNA stability, translation, and splicing.^41^ Pseudouridylation is catalyzed by the PUS family, which serves as the core enzymatic machinery or ‘writers’ essential for understanding the biological significance of this modification.^42^ Increasing evidence suggests that dysfunction of PUS family members, similar to mutations in m6A methyltransferases such as METTL3 and METTL14, is closely associated with tumour initiation, progression, and immune evasion.^43^, ^44^, ^45^, ^46^, ^47^, ^48^, ^49^

Against this backdrop, pan‐cancer integrative analyses at the gene family level have emerged as powerful strategies for identifying key oncogenic drivers. Guan et al. conducted a comprehensive investigation of the DOK gene family across 33 cancer types, revealing its broad involvement in tumour microenvironment remodelling, immune infiltration, and drug sensitivity. Similarly, Lou et al. performed an in‐depth analysis of the PRDM gene family in endometrial cancer, identifying MECOM as a critical oncogene and validating its molecular mechanism.^50^, ^51^ These findings underscore the importance of family‐level analyses in advancing precision oncology and developing targeted therapeutic strategies.

Despite growing interest in RNA modifications, the PUS family has remained comparatively underexplored. Certain members, including DKC1, PUS1, and PUS7, have been implicated in oncogenic processes such as cancer stemness, metabolic reprogramming, and immune regulation across various malignancies.^9^, ^10^, ^11^, ^52^, ^53^, ^54^ We systematically analyzed the expression of the PUS family across multiple cancers based on TCGA data, evaluating TME scores, immune infiltration, cancer stemness, and patient prognosis. We found that several PUS genes are highly expressed in PAAD and are significantly associated with poorer OS and PFS. These characteristics are particularly prominent in PAAD, suggesting that PAAD serves as an important model for elucidating the functional mechanisms of the PUS family. A LASSO regression prognostic model identified PUS3 and PUS7 as significant risk genes, with PUS3 implicated in tRNA‐mediated protein synthesis,^55^ while PUS7 is closely linked to multiple cancers. Furthermore, our study revealed that high PUS7 expression is markedly associated with poor prognosis, enhanced tumour stemness, and reduced immune infiltration, indicating that PUS7 may drive pancreatic cancer progression through modulation of the immune microenvironment.

Within the PUS family, PUS7 emerged as a key candidate in PDAC, a malignancy characterized by dense desmoplasia and profound immune suppression. While previous studies have uncovered a range of functions for PUS7, such as maintaining stemness in glioblastoma via tRNA‐derived fragments,^9^ promoting proliferation and migration in colorectal cancer,^10^, ^11^ and even exerting tumour‐suppressive activity in gastric cancer,^12^ its role in PDAC had not been defined.

Interestingly, our pan‐cancer analyses of PUS family members revealed a highly context‐dependent prognostic impact. Using a PUSs constructed from ssGSEA across TCGA cancers (Figure 3), we observed that high PUSs predicted poor outcomes in tumours with high stemness and immunosuppressive microenvironments, such as PAAD and KIRC, whereas it appeared protective in immune‐infiltrated or low‐stemness tumours, such as LGG and STAD (Figure 3C–F). This context dependency likely arises from tissue‐specific RNA targets, dominant roles of individual PUS members, and modulation by the tumour microenvironment. Specifically, PUS enzymes catalyze pseudouridylation of mRNAs and tRNAs, affecting RNA stability and translation; in pro‐tumourigenic settings, they enhance oncogenic transcripts, whereas in immune‐active tumours, they may stabilize immune‐related RNAs.^56^, ^57^ Furthermore, individual members such as PUS7 in GBM suppress T‐cell recruitment by reducing chemokine expression, promoting immune evasion, while PUS1 in HCC stabilizes proliferation‐related mRNAs, accelerating tumour growth.^57^ Consistently, in PAAD, high PUS7 expression correlated with enhanced stemness, reduced immune infiltration, and poor prognosis, underscoring its role as an independent adverse factor (Figure 4F). Collectively, these findings indicate that the prognostic effects of PUS family members are determined by the balance of specific enzyme expression, RNA targets, and TME interactions rather than total PUS expression alone, highlighting their context‐dependent roles as potential biomarkers and therapeutic targets.^56^, ^57^

Here, we identify a novel function of PUS7 in PDAC, expanding its oncogenic potential beyond intrinsic tumour cell behaviour to include modulation of the tumour immune microenvironment. Mechanistically, our data demonstrate that PUS7 promotes the formation of NETs, chromatin‐based structures extruded by activated neutrophils. Initially recognized for their antimicrobial roles, NETs are now understood to contribute to tumour progression by facilitating metastasis, thrombosis, and immune evasion.^58^, ^59^, ^60^, ^61^, ^62^ In PDAC, recent evidence indicates NETs in sculpting an immunosuppressive TME and driving aggressive tumour behaviour^63^, ^64^, ^65^, ^66^, ^67^. Our findings indicate that PUS7 functions upstream of NETs formation, and that pharmacological degradation of NETs via DNase I abolishes the tumour‐promoting effects of PUS7 in orthotopic mouse models, thereby validating the functional significance of this pathway.

To further analyze the regulatory role of PUS7 within TME and its interaction with NETs, we performed single‐cell RNA sequencing on orthotopic PDAC tumours after adenovirus injection. Consistent with these observations, PUS7 overexpression also promoted tumour growth in vivo. Despite no significant changes in neutrophil abundance, potentially attributable to technical variability during tissue dissociation, PUS7 overexpression was accompanied by diminished infiltration of macrophages and T cells. Specifically, PUS7 overexpression induced a shift towards the M2 macrophage phenotype, which is widely recognized for its immunosuppressive and tumour‐promoting properties.^68^ Importantly, administration of the NETs inhibitor DNase I markedly reversed PUS7‐induced M2 polarization in orthotopic mouse models and significantly suppressed tumour growth in vivo, which suggests that NETs serve as a key mediator in the PUS7‐TAM axis. Previous work has shown that NETs can modulate macrophage behaviour,^23^, ^69^ our findings provide mechanistic insight into how this occurs in PDAC.

The identification of this axis not only deepens our understanding of PDAC immunobiology but also suggests promising therapeutic avenues. Given the profound immune suppression and resistance to immunotherapy that characterize this disease, strategies aimed at remodelling the tumour immune microenvironment are of particular interest. Our findings suggest that targeting PUS7 or downstream NETs formation may represent a viable approach to alleviate immunosuppression and restrain tumour progression.

From a translational perspective, this axis can be therapeutically targeted at multiple levels. Direct inhibition of PUS7 represents an upstream strategy that may simultaneously affect tumour‐intrinsic programs and tumour–immune interactions. In this context, the identification of the small‐molecule PUS7 inhibitor S428‐1145 provides proof‐of‐concept that PUS7 is a druggable RNA‐modifying enzyme. Although further optimization and in vivo validation are required, this approach parallels recent advances in targeting RNA modification machinery, such as METTL3 and FTO, in cancer therapy.^70^, ^71^, ^72^, ^73^, ^74^

Alternatively, downstream targeting of NETs may offer a more immediately actionable strategy. DNase I and other NETs‐disrupting approaches have been explored in preclinical and clinical settings for inflammatory and thrombotic diseases, raising the possibility of repurposing NETs‐targeting agents to modulate the immunosuppressive microenvironment in PDAC. Our data indicate that NETs degradation selectively abrogates PUS7‐driven tumour growth and M2 macrophage polarization, suggesting that NETs inhibition may be particularly effective in tumours with high PUS7 activity.

Notably, emerging evidence indicates that NETs contribute to resistance to both chemotherapy and immune checkpoint blockade by promoting physical barriers, immune exclusion, and suppressive myeloid phenotypes. Therefore, targeting the PUS7–NETs axis may sensitize PDAC tumours to standard‐of‐care chemotherapy or potentially enhance the efficacy of immunotherapies by restoring macrophage polarization and improving immune infiltration. Although such combination strategies remain to be tested, our findings provide a strong rationale for future studies exploring PUS7 or NETs‐targeted interventions in combination with existing treatment modalities. Together, these findings, along with the identification of S428‐1145, support the feasibility of therapeutically targeting the PUS7–NETs axis in PDAC.

Although this study establishes a causal relationship between PUS7 and M2 macrophage polarization mediated by NETs, several limitations remain. The precise molecular mechanisms through which PUS7 regulates NETs formation have not yet been fully elucidated and require further investigation. Additionally, it remains unclear whether the PUS7‐NETs‐macrophage axis is conserved across other tumour types. A comprehensive analysis of the upstream regulatory network of PUS7, along with the identification of its pseudouridylation targets, may provide novel mechanistic insights and inform the development of targeted therapeutic strategies.

In summary, we elucidated the association between the PUS family and tumour progression, with a particular focus on PUS7 in pancreatic ductal adenocarcinoma PDAC. We demonstrated that PUS7 promotes PDAC growth and metastasis, partly through its regulation of NETs and TAM polarization. This study defines a novel immunoregulatory axis, PUS7‐NETs‐TAMs, in PDAC. Furthermore, we identified S428‐1145 as a potential PUS7 inhibitor, highlighting PUS7 as a promising therapeutic target and providing mechanistic insights and a foundation for future drug development.

## AUTHOR CONTRIBUTIONS

Jike Fang performed the experiments. Shiye Ruan, Yajie Wang and Yue Chen collected and analyzed the data. Baohua Hou, Shanzhou Huang and Chuanzhao Zhang designed the study. Fuxin Huang and Zhongyan Zhang contributed to the revision. Jike Fang and Shiye Ruan wrote the manuscript. All authors read and approved the final manuscript.

## CONFLICT OF INTEREST STATEMENT

The authors declare no conflict of interest.

## FUNDING INFORMATION

This study was supported by the National Natural Science Foundation of China (8210101087 and 82573899), Basic and applied basic research funding Guangdong Province (2025A1515012422), the Science and Technology Program of Maoming (2024kjcxLX046), Wu Jieping Medical Foundation (320.6750.2025‐18‐11), Young Talent Support Project of Guangzhou Association for Science and Technology (QT2024‐037).

## ETHICS APPROVAL AND CONSENT TO PARTICIPATE

In this study, clinical data from 56 patients with pancreatic cancer were collected for expression and survival analyses. All pancreatic cancer specimens were pathologically confirmed by expert pathologists. The study protocol was approved by the Ethics Committee of Guangdong Provincial People's Hospital (Institutional Review Board), approval number KY2025‐648‐01. Informed consent was obtained from all patients. All relevant animal experimental procedures were approved by the Animal Care and Use Committee of Guangzhou Lingfu Tuopu Biotechnology Company Limited (ethics approval ID: G2023‐287‐01).

## CONSENT FOR PUBLICATION

Not applicable.

## Supporting information



Supporting Information

## Data Availability

The datasets used and analyzed during the current study are available from the corresponding author on reasonable request.
