# Supplementary material for: Multi‐omics analysis identifies PUS7 as an immune modulator driving NETs‐mediated macrophage polarization in pancreatic cancer
Source: Clin Transl Med. 2026 Jan 6;16(1):e70581. doi: 10.1002/ctm2.70581 (PMC12775584; doi:10.1002/ctm2.70581)
Supplement: Supplementary file 1 — Supporting Information [file CTM2-16-e70581-s001.docx]

**Multi-Omics Analysis Identifies PUS7 as an Immune Modulator Driving NETs-Mediated Macrophage Polarization in Pancreatic Cancer**

Jike Fang ^a, b, 1^, Shiye Ruan ^a, d, 1^, Yajie Wang ^a, c, 1^, Yue Chen ^a, b, 1^, Fuxin Huang ^a, c^, Zhongyan Zhang ^a, b^, Chuanzhao Zhang ^a, b, c,^ ^*^, Baohua Hou ^a, e, *^, Shanzhou Huang ^a, b, c,^ ^**^.

^a^ Department of General Surgery, Guangdong Provincial People’s Hospital (Guangdong Academy of Medical Sciences), Southern Medical University, Guangzhou 510080, China.

^b^ The Second School of Clinical Medicine, Southern Medical University, Guangzhou, Guangdong 510515, China.

^c^ South China University of Technology School of Medicine, Guangzhou, Guangdong 510006, China.

^d^ Division of Hepatobiliary and Pancreas Surgery, Department of General Surgery, Shenzhen People's Hospital (The Second Clinical Medical College, Jinan University; The First Affiliated Hospital, Southern University of Science and Technology), 518020 Shenzhen, China.

^e^ Maoming People’s Hospital, Maoming, Guangdong 525000, China.

^*^ Corresponding author.

^*^ Corresponding authors at: Department of General Surgery, Guangdong Provincial People’s Hospital, Guangdong Academy of Medical Sciences, Southern Medical University, Guangzhou, Guangdong 510080, China.

^**^ Corresponding author at: Department of General Surgery, Guangdong Provincial People’s Hospital (Guangdong Academy of Medical Sciences), Southern Medical University, Guangzhou 510080, China, Maoming People’s Hospital, Maoming, Guangdong 525000, China.

E-mail addresses: zhangchuanzhao@gdph.org.cn (C. Zhang), huangshanzhou@gdph.org.cn (S. Huang), hbh1000@126.com (B. Hou).

^1^ These authors contributed equally to this work.

**Supplementary Figures**

**
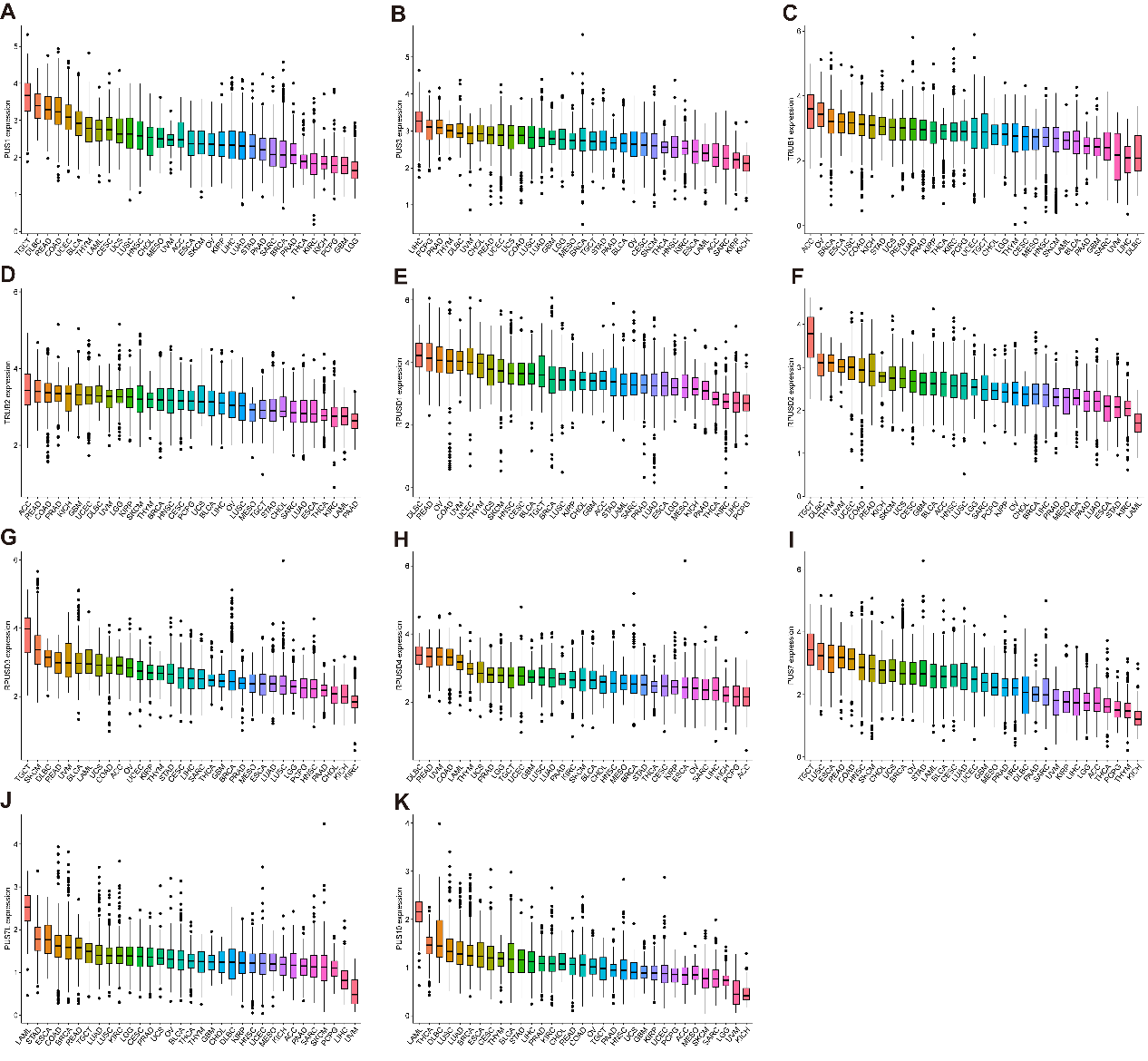
**

**Figure S1. The link of each PUS family member with 33 types of pan-cancer utilizing data from the TCGA database.** A-K. The expression of all PUS family gene in all types of TCGA database.


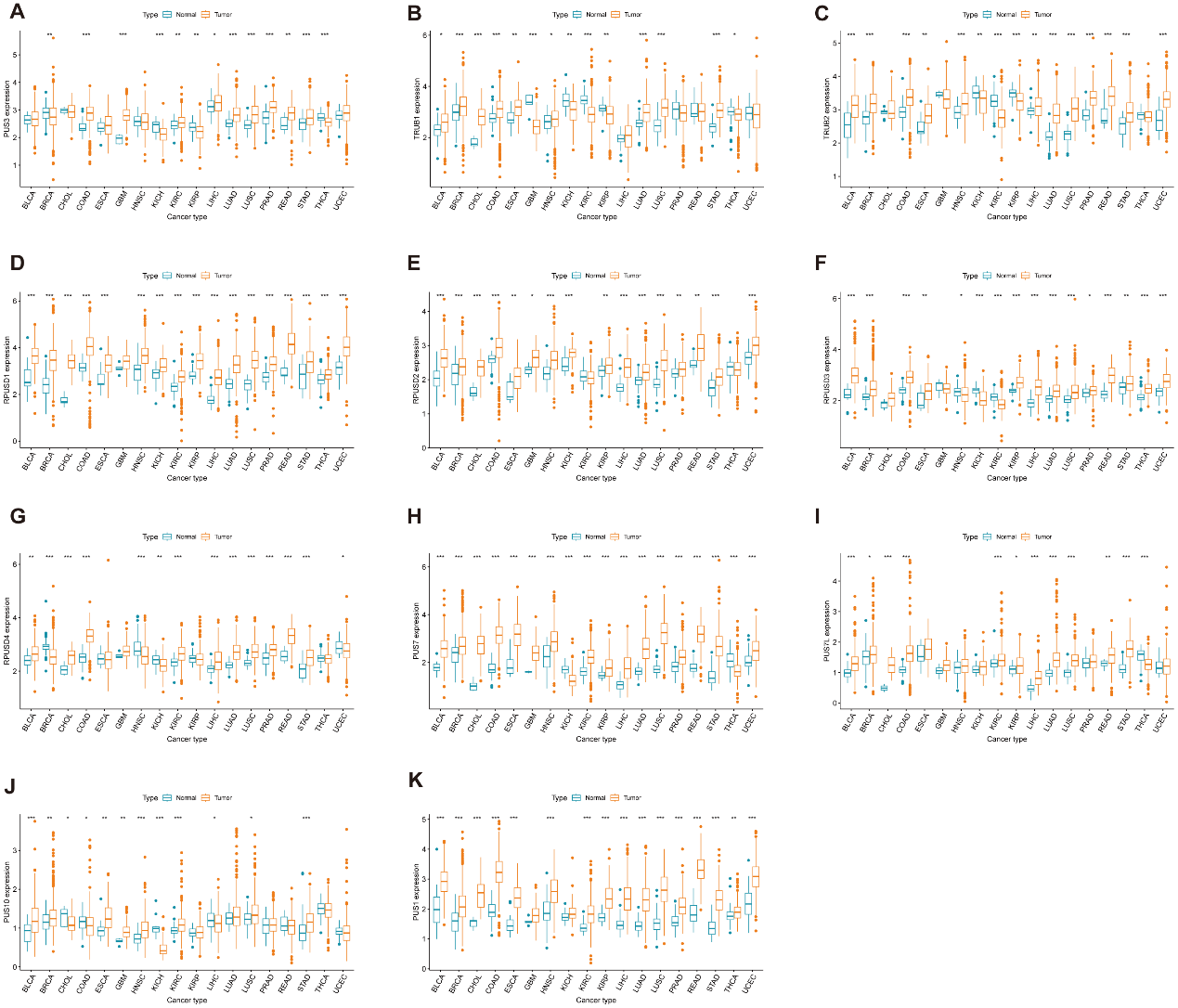


**Figure S2. Differential expression between cancer and adjacent normal tissues.** A-K. Differential expression of all PUS family gene in cancer (red boxplots) and adjacent normal tissues (blue boxplots). *P < 0.05, **P < 0.01, ***P < 0.001.


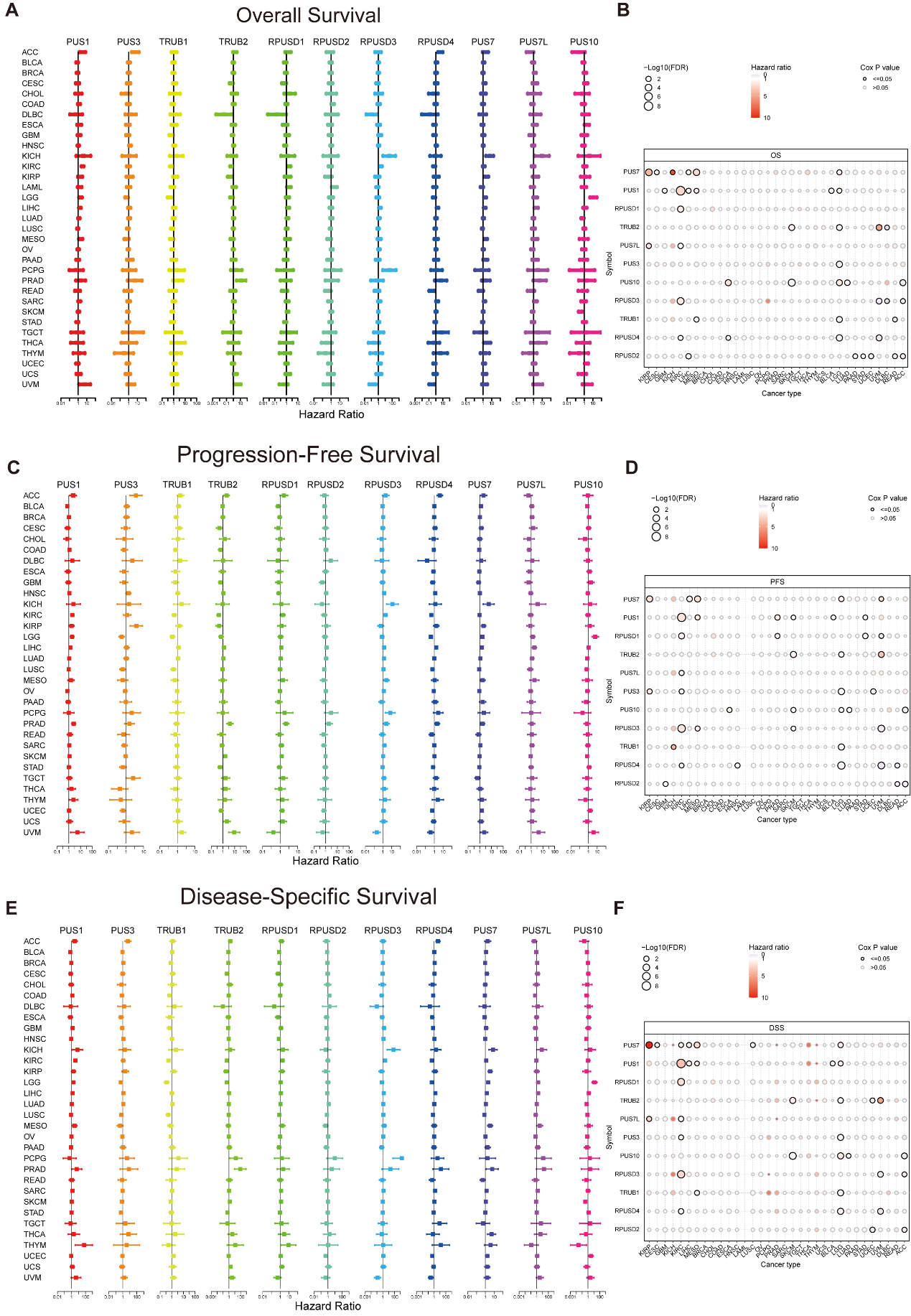


**Figure S3. The survival analysis of PUS score.** A–F. Forest plots of the Cox analysis results of PUS family genes in pan-cancer. Overall survival (A, B), progression-free interval (C, D), disease-specific survival (E, F).


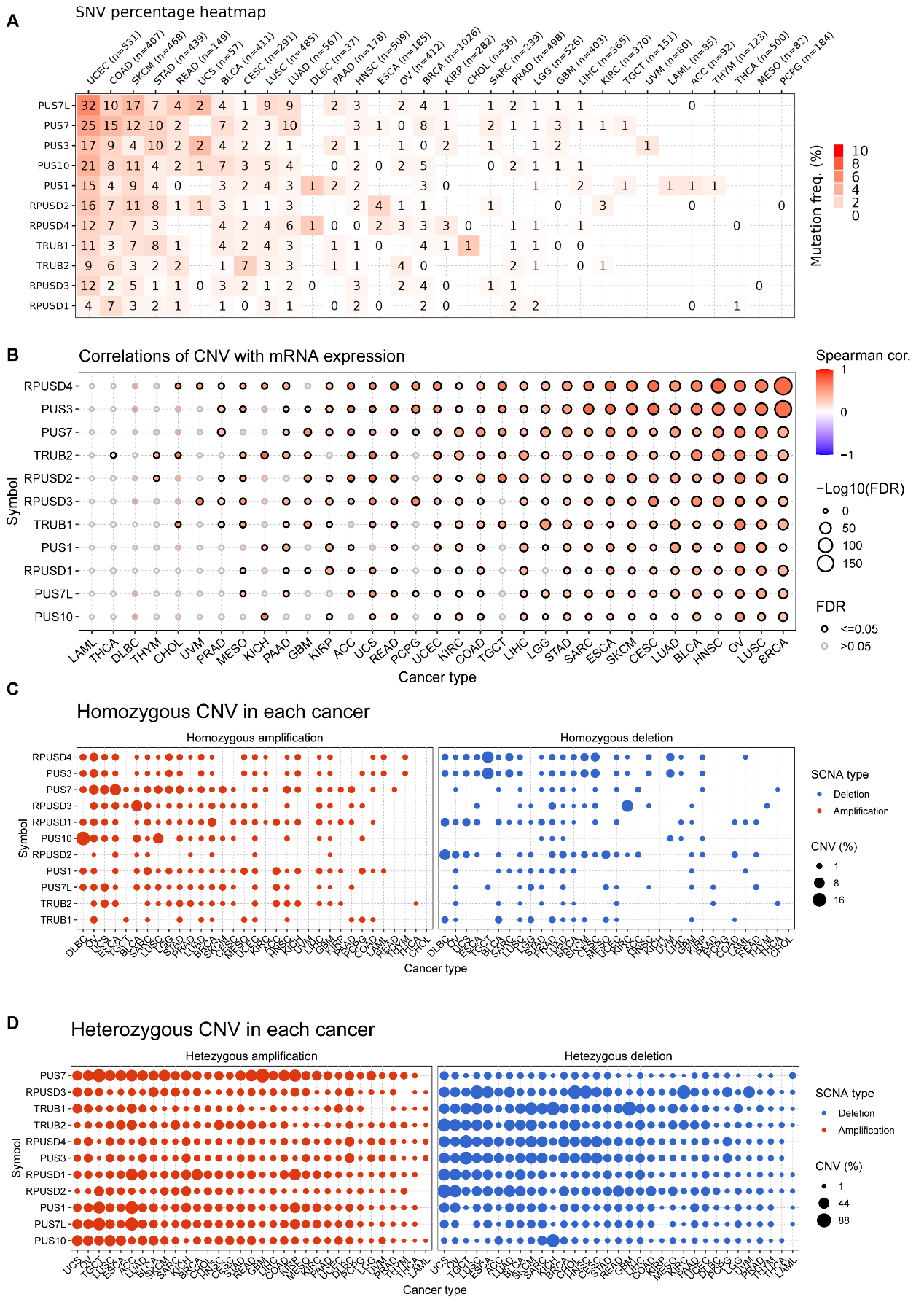


**Figure S4. Gene alterations in PUS family genes across 33 cancer types.** A. The frequency landscape of deleterious mutations in 33 tumor types. B. The relationship of CNV with the mRNA levels of PUS family genes. C-D. The percentage of homozygous (C) or heterozygous (D) CNV (including homozygous amplification, homozygous deletion, heterozygous amplification, and heterozygous deletion) for each PUS member in pan-cancer.


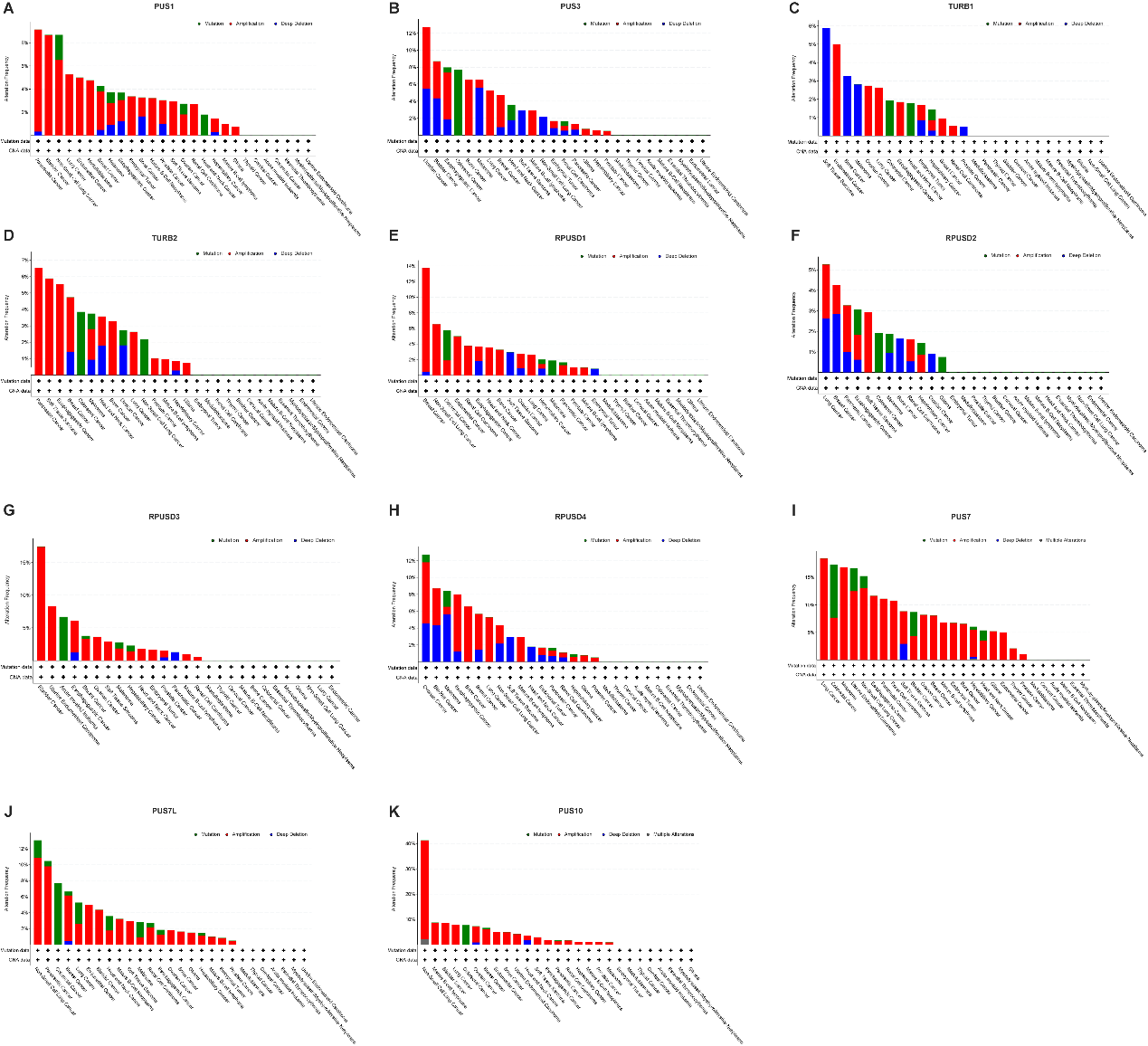


**Figure S5. The mutation rate of individual PUS genes.** A-K. in the context of pan-cancer analysis and specific cancer subtypes, utilizing the ICGC/TCGA pan-cancer datasets (comprising 2683 samples) via cBioPortal.


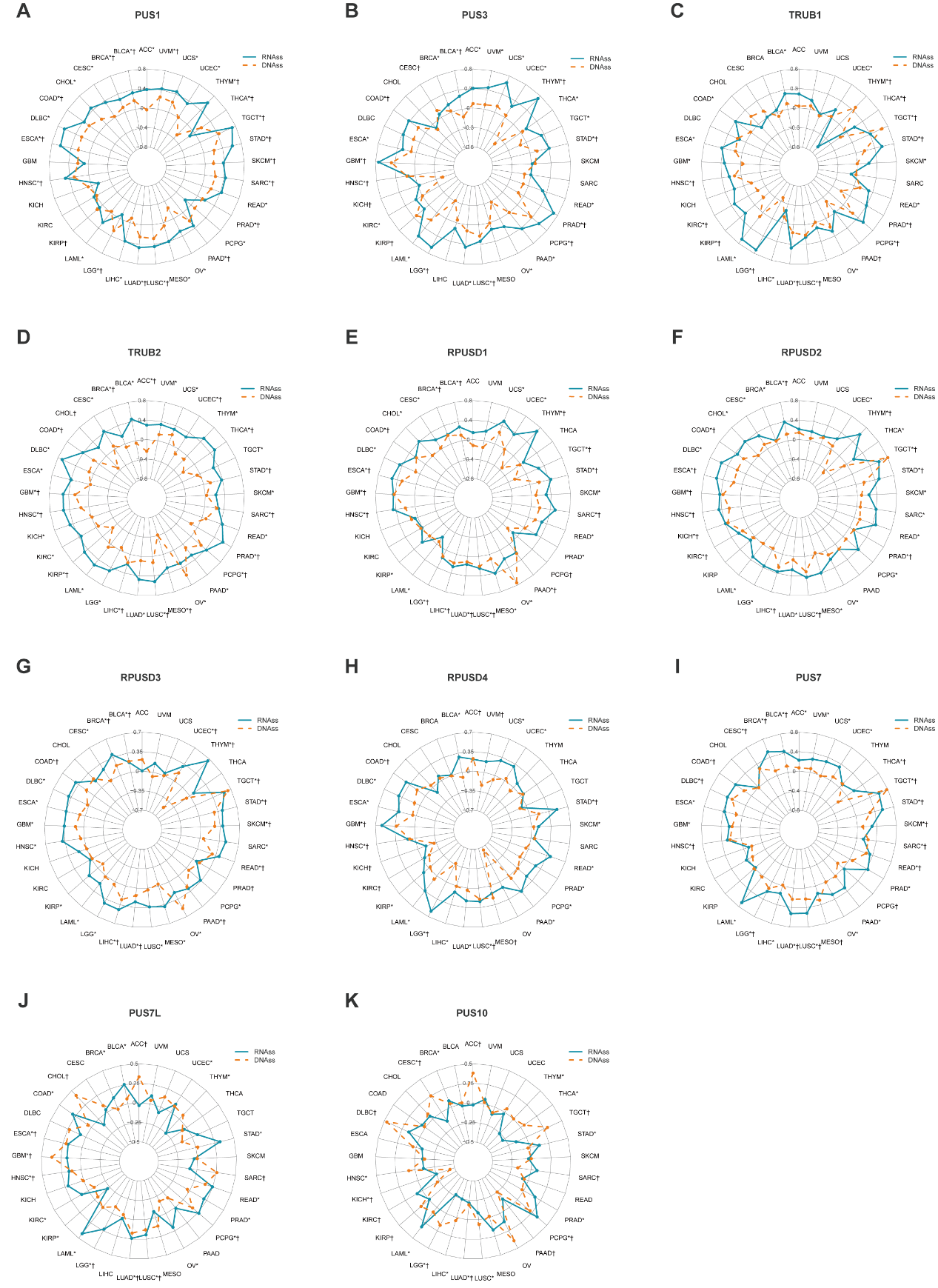


**Figure S6. RNAss and DNAss Correlation Analysis.** A-K. The radar chart of RNAss and DNAss in TCGA database. † DNAss; * RNAss. *P < 0.05, **P < 0.01, ***P < 0.001.

**
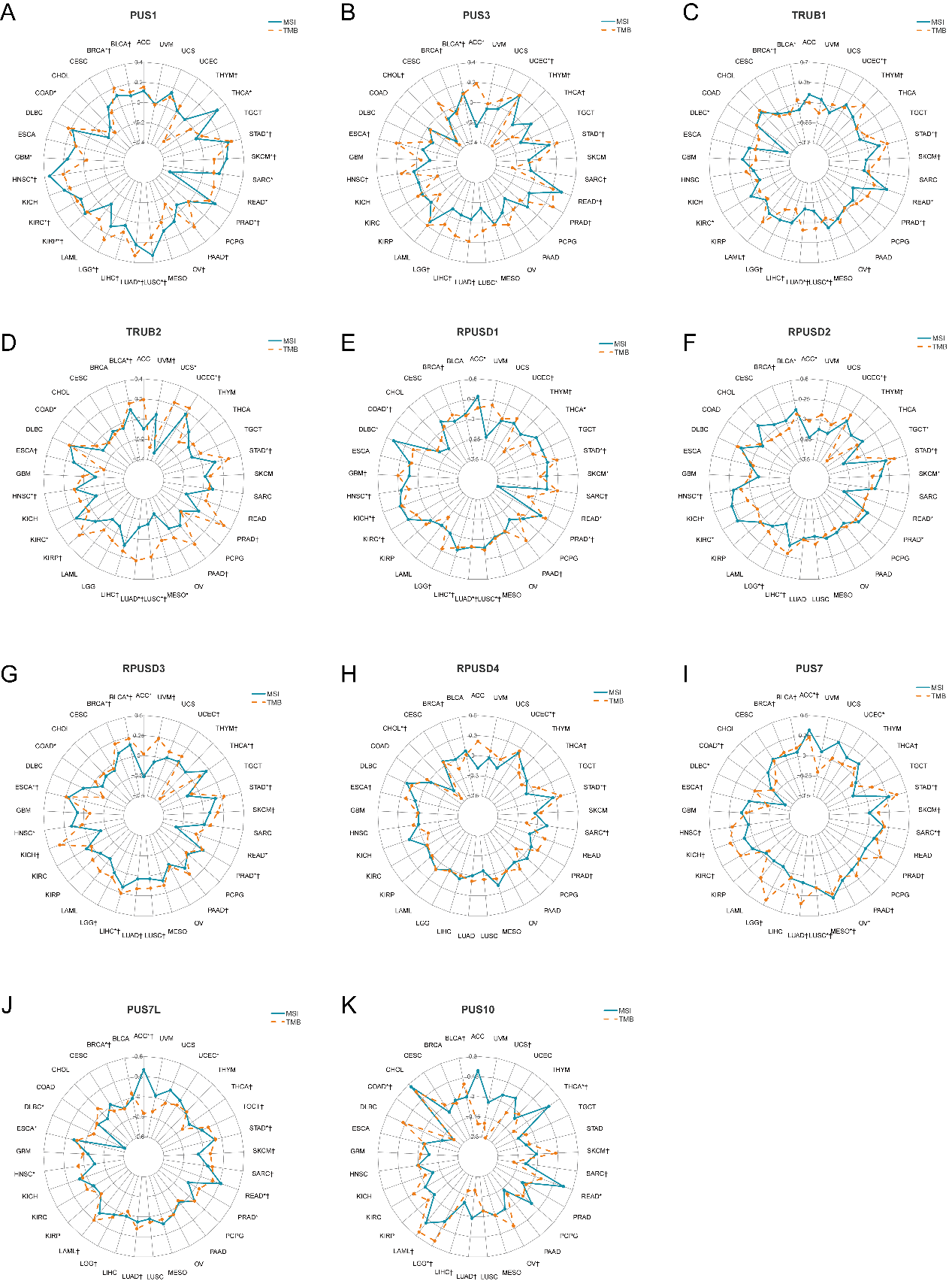
**

**Figure S7. Correlation analysis of diverse PUS family genes expression with TMB and MSI.** The radar chart of TMB and MSI score in TCGA database. † TMB; * MSI. *P < 0.05, **P < 0.01, ***P < 0.001.


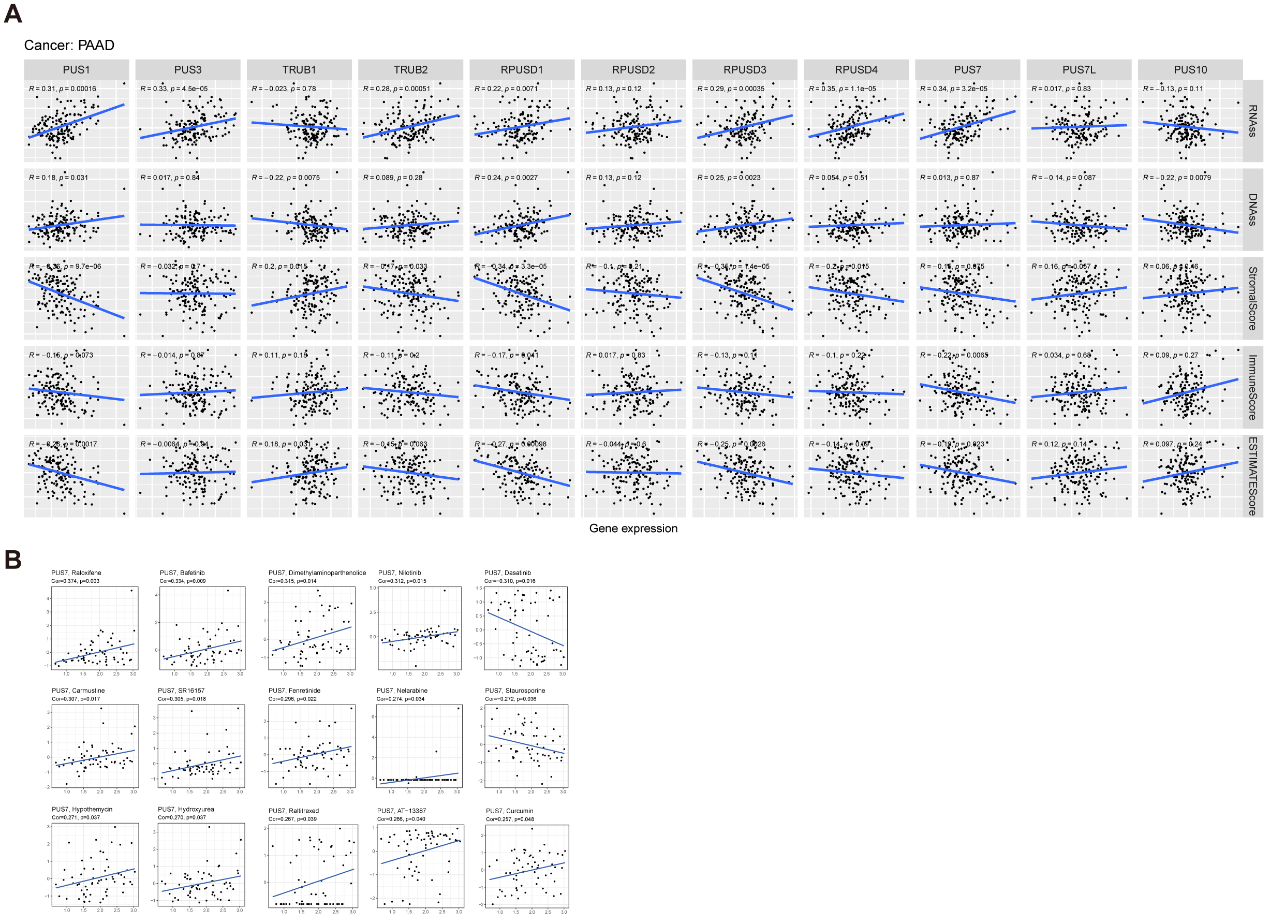


**Figure S8. The expression patterns of PUS family genes and the potential relationship between PUS7 expression and drug sensitivity.** A. The association between PUS family genes expression and stemness indices (RNAss and DNAss), stromal scores, immune scores, and ESTIMATE scores in PAAD in TCGA cancer. B. Top 15 drugs and their significant associations with PUS7 (P < 0.05).

**
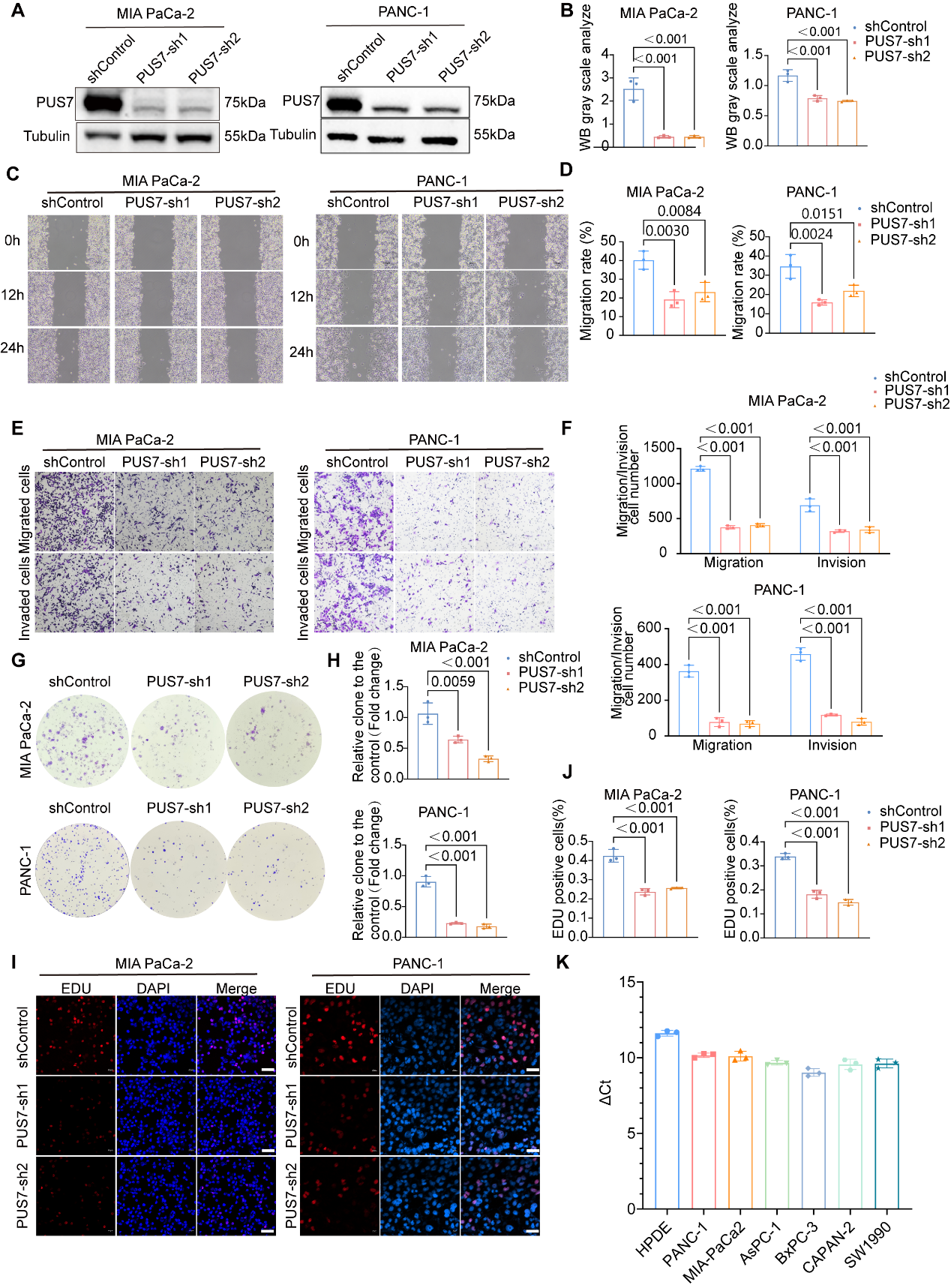
**

**Figure S9. PUS7 promotes proliferation, migration, and invasion of pancreatic cancer cells.** A-B. Western blot analysis confirming the knockdown efficiency of PUS7 in PANC-1 and MIA PaCa-2 cells. Data are mean ± SEM (n = 3 independent experiments); unpaired two-tailed Student’s t-test. C-D. Wound healing assays evaluating the effect of PUS7 knockdown on cell migration. Data are mean ± SEM (n = 3 independent experiments); unpaired two-tailed Student’s t-test. E-F. Transwell invasion assays assessing the invasive capabilities of PDAC cells transfected with sh-control or PUS7-shRNA. Bar graphs represent the relative number of cells that invaded through the membrane (normalized to the control group). Data are mean ± SEM (n = 3 independent experiments); unpaired two-tailed Student’s t-test. G-H. Colony formation assays measuring the proliferative capacity of PANC-1 and MIA PaCa-2 cells. Data are mean ± SEM (n = 3 independent experiments); unpaired two-tailed Student’s t-test. I-J. EdU incorporation assays evaluating cell proliferation in PANC-1 and MIA PaCa-2 cells. Scale bar = 50μm. Data are mean ± SEM (n = 3 independent experiments); unpaired two-tailed Student’s t-test. K. Complete distribution of raw Ct values underlying the qRT-PCR results presented in Figure 5I. Data are presented as mean ± SEM (n=3 per group). Statistical significance was assessed using one-way ANOVA followed by LSD or Dunnett’s T3 multiple comparisons test.


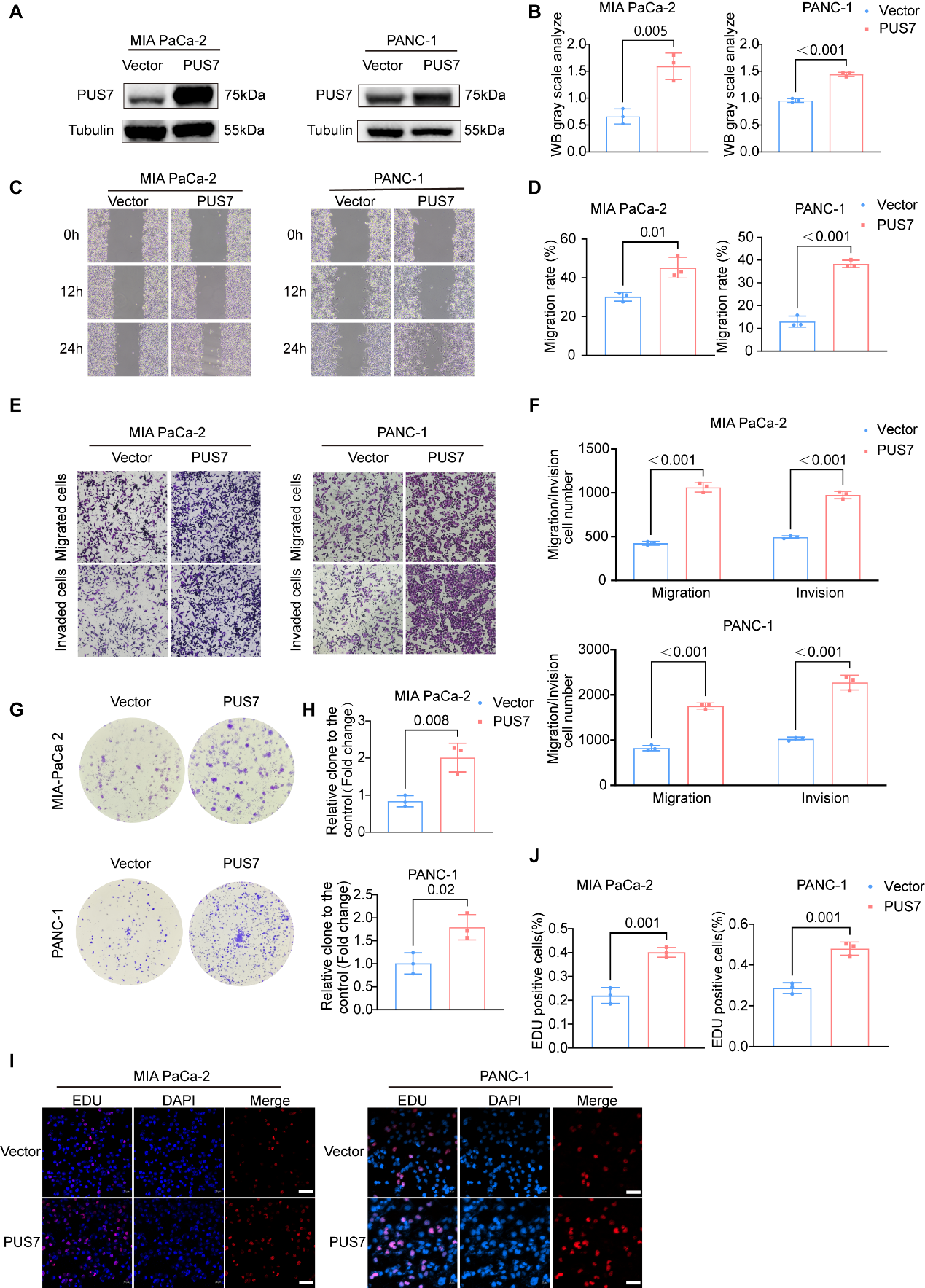


**Figure S10. PUS7 overexpression promotes the proliferation, migration, and invasion of pancreatic cancer cells.** A-B. Western blot analysis validating the overexpression efficiency of PUS7 in PANC-1 and MIA PaCa-2 cells. Data are mean ± SEM (n = 3 independent experiments); unpaired two-tailed Student’s t-test. C-D. Wound healing assays assessing the migratory capacity of PANC-1 and MIA PaCa-2 cells upon PUS7 overexpression. Data are mean ± SEM (n = 3); unpaired two-tailed Student’s t-test. E-F. Transwell migration and invasion assays performed in PANC-1 and MIA PaCa-2 cells following PUS7 overexpression. Data are mean ± SEM (n = 3); unpaired two-tailed Student’s t-test. G-H. Colony formation assays evaluating the proliferative potential of PANC-1 and MIA PaCa-2 cells with PUS7 overexpression. Data are mean ± SEM (n = 3); unpaired two-tailed Student’s t-test. I-J. EdU incorporation assays conducted in PUS7-overexpressing PANC-1 and MIA PaCa-2 cells to determine the percentage of S-phase cells (EdU, red; Hoechst, blue; representative of three independent experiments). Scale bar = 50 μm. Hoechst staining indicates total cell nuclei, while EdU incorporation reflects active DNA replication. Data are mean ± SEM (n = 3); unpaired two-tailed Student’s t-test.


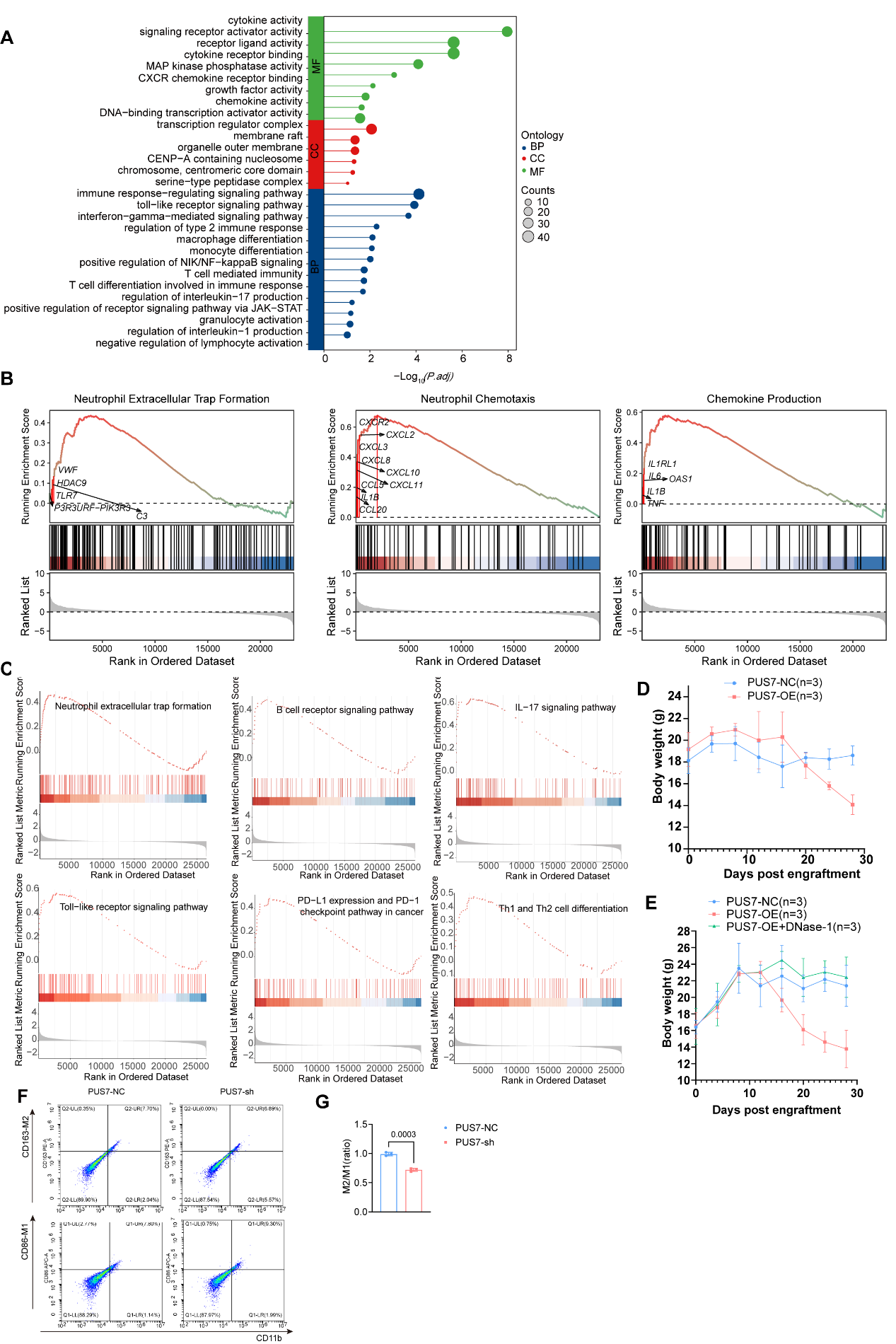


**Figure S11. PUS7 promotes the formation of neutrophil extracellular trap (NET)-forming cells in pancreatic cancer.** A. GO enrichment dot plot showing differentially expressed genes between the PUS7-NC and PUS7-OE groups. Statistical analysis performed using Fisher’s exact test for GO term enrichment. B. PUS7-regulated tumor cell–secreted factors associated with NET formation. RNA-seq analysis identified CXCL2, CXCL8, and IL-6 as upregulated by PUS7 overexpression, suggesting a role in promoting neutrophil activation and NETosis. C. GSEA analysis identifying immune-related pathway differences between PUS7-NC and PUS7-OE groups. D. Longitudinal body weight changes of mice during tumor growth. PUS7-OE tumor-bearing mice showed earlier and greater weight loss than controls, reflecting increased systemic tumor burden. Data are mean ± SEM (n = 3 per group). E. Body weight of mice showed dynamic changes during tumor growth. Compared to the control group, PUS7-OEtumor-bearing mice exhibited earlier onset and greater magnitude of body weight loss, reflecting increased systemic tumor burden; in contrast, DNAse-1 treated tumor-bearing mice did not show significant weight loss, with body weight similar to that of the NC group. Data are presented as mean ± SEM (n = 3 per group). F. Representative flow cytometry plots showing the expression of M1 (CD86) and M2 (CD206) markers. G. Quantification of the M2/M1 macrophage ratio. PUS7 knockdown significantly reduced M2 polarization. Data are presented as mean ± SEM; n = 3 independent experiments.


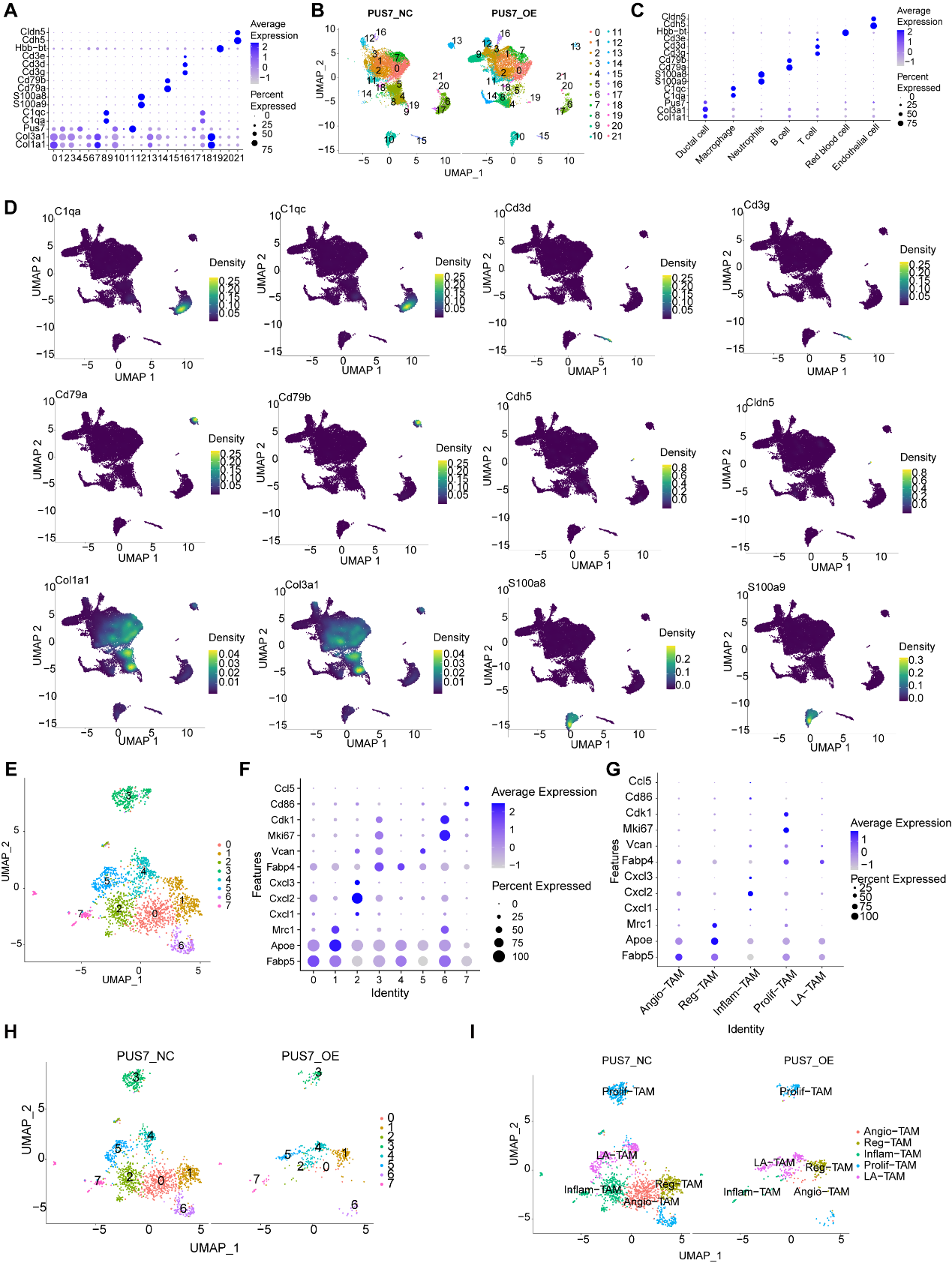


**Figure S12. Single-cell transcriptomic analysis reveals the regulatory effects of PUS7 on the tumor immune microenvironment in pancreatic cancer.** A. Bubble plot displaying the expression of marker genes across 22 identified cell clusters. B. Pancreatic cancer cells were stratified into two groups based on PUS7 expression levels. C. Bubble plot illustrating the marker gene expression profiles of various cell types. D. UMAP plots showing marker gene expression in specific cell types in PUS7 control and PUS7-overexpressing groups: pancreatic ductal cells (Col1a1, Col3a1), macrophages (C1qc, C1qa), T cells (Cd3e, Cd3d, Cd3g), B cells (Cd79a, Cd79b), neutrophils (S100a8, S100a9), and endothelial cells (Cldn5, Cdh5). E. UMAP plot displaying eight distinct macrophage clusters identified by dimensionality reduction. F. Bubble plot showing expression of marker genes within each macrophage subcluster. G. Bubble plot presenting the expression of specific marker genes for different macrophage subtypes. H-I. UMAP plots illustrating the distribution of macrophage populations in the PUS7 control group (left) and the PUS7-overexpression group (right), respectively.

**
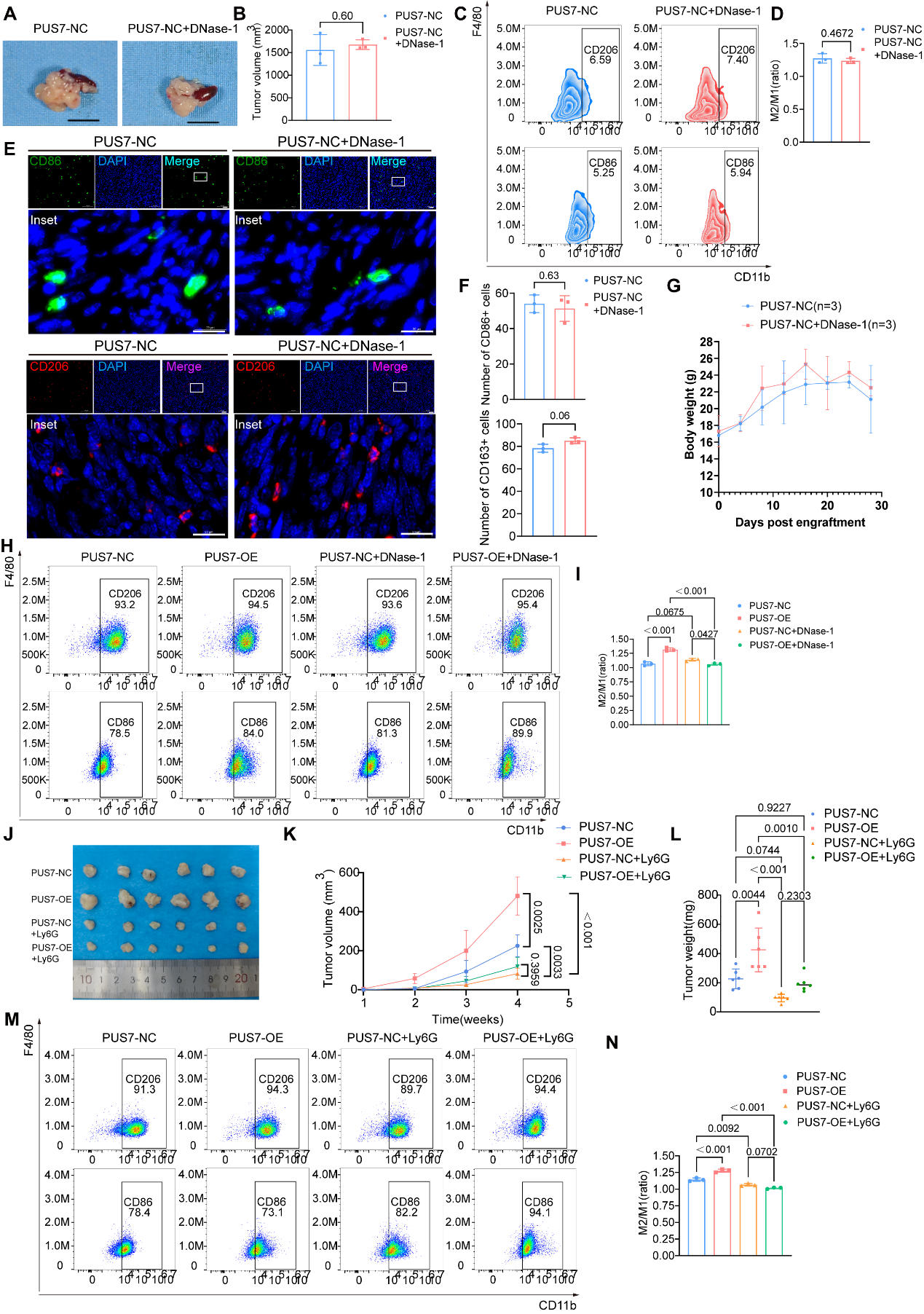
Supplementary Figure S13. Context-dependent effects of NETs degradation and neutrophil depletion on PUS7-driven tumor progression and macrophage polarization.**
A-B. Tumor growth curves (A) and endpoint tumor volumes (B) of mice bearing Vector control tumors treated with vehicle or DNase I. C-F. Flow cytometric analysis of tumor‐infiltrating macrophage populations showing no significant differences in macrophage polarization between groups. G. Body weight monitoring of mice during DNase I treatment. Data are shown as mean ± SEM; ns, not significant. H. Schematic illustration of the Transwell-based sequential co-culture system used to assess NETs-mediated macrophage polarization. Neutrophils were stimulated with conditioned medium from Pan02-PUS7 cells to induce NETs formation, followed by DNase I treatment to degrade NETs before macrophage exposure. I. Flow cytometric analysis of macrophage polarization following treatment with NETs-containing supernatant, shown as the CD206/CD86 ratio. Data are presented as mean ± SEM from independent experiments and analyzed by one-way ANOVA. J-K. Tumor growth curves (J) and endpoint tumor volumes (K) in mice bearing PUS7-overexpressing tumors treated with isotype control or anti-Ly6G antibody. L-N. Flow cytometric analysis (L) and quantification (M–N) of macrophage polarization in tumors following neutrophil depletion. Data are shown as mean ± SEM and analyzed using unpaired two-tailed Student’s *t*-test.


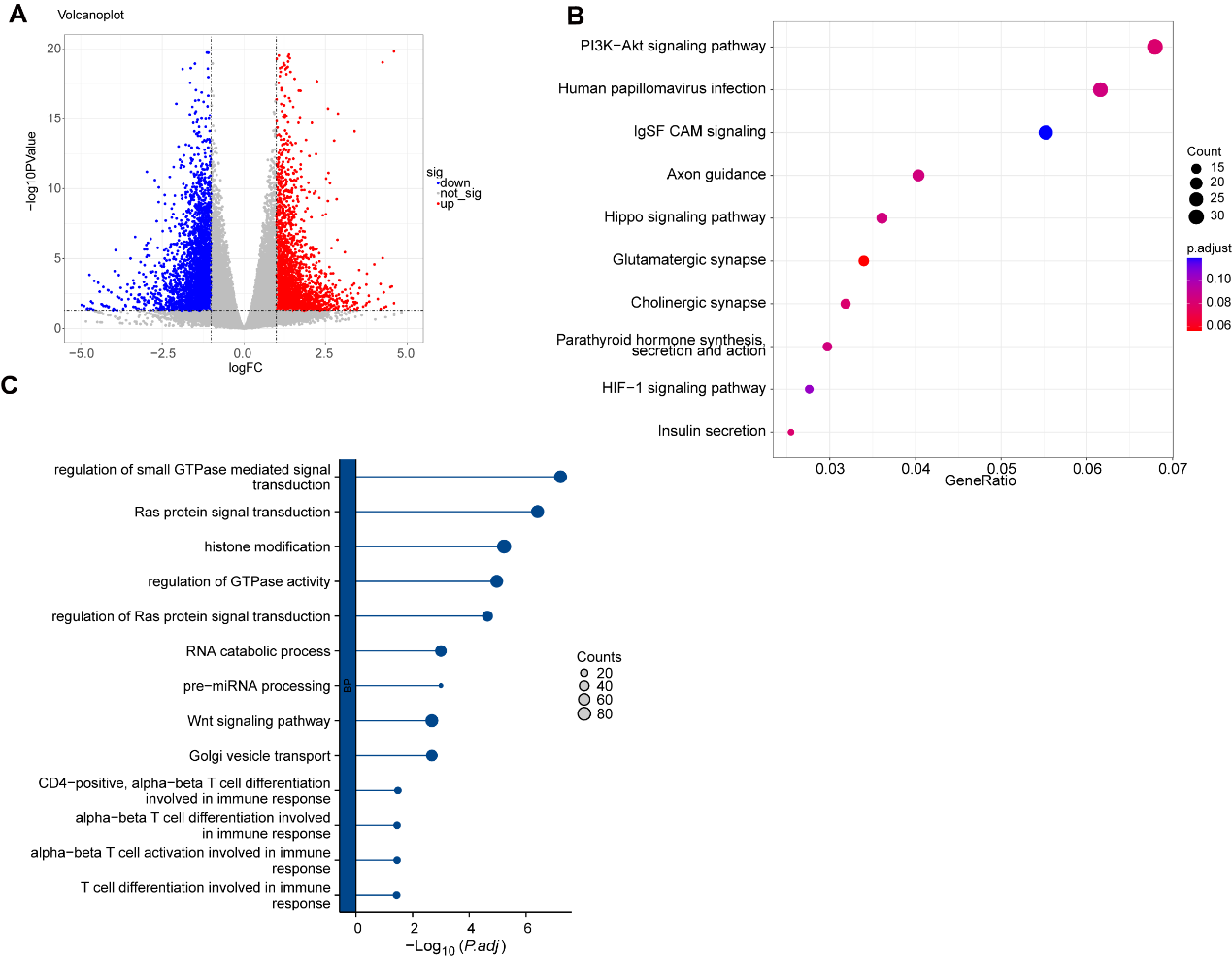


**Supplementary Figure S14. PUS7 RNA targets identified by RIP-seq.**

A. Volcano plot showing differentially enriched mRNAs in PUS7-overexpressing (OE) versus control (NC) cells, highlighting selective RNA binding by PUS7.

B–C. Gene Ontology (B) and KEGG pathway (C) enrichment analyses of PUS7-bound mRNAs identified by RIP-seq, highlighting pathways related to oncogenic signaling and immune regulation, including PI3K–Akt, Ras, Hippo, Wnt, HIF-1 signaling, and T-cell differentiation and activation.

**Supplementary Tables**

**Supplementary Table S1. The core interference sequences of shRNAs.**

| Name | Sequence |
| --- | --- |
| Sh1-PUS7 | CGTTGCATTGATAGGCCCATT |
| Sh2-PUS7 | GCCTACCGAAAGATCATTATT |

**Supplementary Table S2. Primer sequences for real-time RT-PCR**

| Gene | Sequence (5’-3’) |
| --- | --- |
| PUS7-F | GACATGATGAAGCATGGACTCA |
| PUS7-R | CATGAACAACGAAGTCGGAGTAT |
| GAPDH-F | GGAGCGAGATCCCTCCAAAAT |
| GAPDH-R | GGCTGTTGTCATACTTCTCATGG |

**Supplementary Table S3. List of primary antibodies**

| Antibody | Host | Cat. no | Supplier | Dilution | Use for |
| --- | --- | --- | --- | --- | --- |
| PUS7  MPO  Histone H3 | Mouse  Rabbit  Rabbit | MG73185S  22225-1-AP  17168-1-AP | Abmart  Prteintech  Prteintech | 1:5000  1:1000  1:10,000 | WB  WB  WB |
| HRP-  αTubulin | Rabbit | HRP-66031 | Prteintech | 1:10,000 | WB |
| PUS7 | Rabbit | ab224119 | Abcam | 1:200 | IHC |
| Histone H3 | Rabbit | ab5103 | Abcam | 1:100 | IF、FC |
| MPO | Rabbit | ab208670 | Abcam | 1:100 | IF、FC |
| NE  F4/80  CD11b  Ly-6G  CD45  CD206  CD86  CD163  CD86 | Rabbit  Mouse  Mouse  Mouse  Mouse  Mouse  Mouse  Human  Human | ab310335,  157317  101205  127613  157207  141707  159203  326505  374207 | Abcam  BioLegend  BioLegend  BioLegend  BioLegend  BioLegend  BioLegend  BioLegend  BioLegend | 1:2000  1:100  1:100  1:100  1:100  1:100  1:100  1:100  1:100 | FC  FC  FC  FC  FC  FC  FC  FC  FC |
| CD11b | Rabbit | 301330 | Proteintech | 1:2000 | FC |

**Supplementary Table S4. List of secondary antibodies**

| Target | Source | Conjugated | Cat. no | Supplier | Dilution | Use for |
| --- | --- | --- | --- | --- | --- | --- |
| anti-rabbit IgG(H+L)  anti-mouse  IgG(H+L) | Goat  Goat | HRP  HRP | SA00001-2  SA00001-1 | Proteintech  Proteintech | 1:10,000  1:10,000 | WB  WB |
| anti-Mouse/Rabbit | Goat | HRP-Polymer | KIT-5020 | MaxVision | - | IHC |
| Anti-Rabbit IgG H&L (PE) | Goat | - | ab72465 | Abcam | 1:100 | FC |

HRP, horseradish peroxidase; IHC, immunohistochemistry; WB, western blottin; FC, Flow Cytometry

**Supplementary Table S5.** **The staining scoring criteria.**

| Score | 0 | 1 | 2 | 3 | 4 |
| --- | --- | --- | --- | --- | --- |
| Extent | 1%-4% | 5%-25% | 26%-50% | 51%-75% | >75% |
| Intensity | no staining | Weak staining | intermediate staining | strong staining | - |

*The multiple of the intensity and extent score was used as the final IHC score (0-12).
